# A Pilot RCT of Psychodynamic Group Art Therapy for Patients in Acute Psychotic Episodes: Feasibility, Impact on Symptoms and Mentalising Capacity

**DOI:** 10.1371/journal.pone.0112348

**Published:** 2014-11-13

**Authors:** Christiane Montag, Laura Haase, Dorothea Seidel, Martin Bayerl, Jürgen Gallinat, Uwe Herrmann, Karin Dannecker

**Affiliations:** 1 Charité University Medicine Berlin (Charité Universitätsmedizin Berlin), Department of Psychiatry and Psychotherapy, Campus Mitte, Berlin, Germany; 2 Weissensee School of Art (Kunsthochschule Berlin-Weißensee), Art Therapy Department, Berlin, Germany; Shanghai Mental Health Center, Shanghai Jiao Tong University School of Medicine, China

## Abstract

This pilot study aimed to evaluate the feasibility of an assessor-blind, randomised controlled trial of psychodynamic art therapy for the treatment of patients with schizophrenia, and to generate preliminary data on the efficacy of this intervention during acute psychotic episodes. Fifty-eight inpatients with DSM-diagnoses of schizophrenia were randomised to either 12 twice-weekly sessions of psychodynamic group art therapy plus treatment as usual or to standard treatment alone. Primary outcome criteria were positive and negative psychotic and depressive symptoms as well as global assessment of functioning. Secondary outcomes were mentalising function, estimated with the Reading the mind in the eyes test and the Levels of emotional awareness scale, self-efficacy, locus of control, quality of life and satisfaction with care. Assessments were made at baseline, at post-treatment and at 12 weeks' follow-up. At 12 weeks, 55% of patients randomised to art therapy, and 66% of patients receiving treatment as usual were examined. In the per-protocol sample, art therapy was associated with a significantly greater mean reduction of positive symptoms and improved psychosocial functioning at post-treatment and follow-up, and with a greater mean reduction of negative symptoms at follow-up compared to standard treatment. The significant reduction of positive symptoms at post-treatment was maintained in an attempted intention-to-treat analysis. There were no group differences regarding depressive symptoms. Of secondary outcome parameters, patients in the art therapy group showed a significant improvement in levels of emotional awareness, and particularly in their ability to reflect about others' emotional mental states. This is one of the first randomised controlled trials on psychodynamic group art therapy for patients with acute psychotic episodes receiving hospital treatment. Results prove the feasibility of trials on art therapy during acute psychotic episodes and justify further research to substantiate preliminary positive results regarding symptom reduction and the recovery of mentalising function.

**Trial Registration:**

ClinicalTrials.gov NCT01622166

## Introduction

Art therapy (AT) has a long tradition for the understanding and the treatment of patients with severe mental disorders [1]–[4], and group art therapy has become a common adjunctive intervention in hospital settings [5]. After early attempts to use the artifacts of psychiatric patients as a diagnostic medium and to gain access to their unconscious inner life, contemporary art therapy is valued as a non-verbal, ressource-oriented “form of therapy in which the making of visual images… in the presence of a qualified art therapist contributes towards externalisation of thoughts and feelings which may otherwise remain unexpressed” [6]. The development of art therapy was inextricably linked to the treatment of psychotic patients, which on the one hand can be seen as a consequence of the long-standing interest in their creative expressions [7], but on the other must be related to the unique properties of art therapy itself, rendering it perhaps more suitable for acutely psychotic persons than other forms of exploratory psychotherapy [8]. The creative space offers the opportunity to express and communicate even unusual experiences in a validating and non-judgmental environment. The tripartite relationship between the patient/creator, the therapist and the artifact allows a finely nuanced regulation of interpersonal contact and intensity [9]. Art therapy has been considered particularly helpful to patients who are unable to express their inner life verbally - may it be because of its 'abnormal' or 'unspeakable' contents [9], formal thought disorder, alexithymia [10], or fear of being overwhelmed by emotion [3]. Moreover, creating artworks may affirm the vulnerable sense of self and ownership in psychoses [8], [11], foster benign self-awareness [12], [13] and strengthen self-efficacy in the process of recovery [14]. Art therapy in psychotic people can help to restore orientation and structure within internal and external experiences, and the artistic 'externalisation' of inner states or experiences can become subject to cautious distancing and reality-testing [4]. In contrast to normal symbol-formation, images in acute schizophrenia may not always have an 'as-if' quality. This may correspond to the large body of evidence reporting a reduced mentalising capacity, or deficits in theory of mind, empathy and the understanding of metaphor in this disorder [15]–[19]. Previous findings suggest that mentalising deficits are detectable in all stages and the entire spectrum of psychotic disorders, but underlie substantial alterations during acute phases of illness [20]. Art therapy might, like any other psychotherapy, but even more so through the reflection of images and artifacts, help to recover mentalising function in psychosis by promoting the formation of mental representations of thoughts and feelings and to develop a “language for mental content which supports mentalisation” [13]. To jointly reflect about and to share the experience of art can invite to adopt the perspective of others [4].

Under the umbrella of creative therapies and based on a meta-analysis compassing art, music, drama and dance movement therapies, art therapy was integrated in international treatment recommendations for schizophrenia like the British NICE guidelines [21]. The recently issued guideline on psychosocial therapies in severe mental disorders of the German Society for Psychiatry, Psychotherapy and Neurology endorsed art therapy with a recommendation grade B [22]. However, compared to the wealth of clinical experience, naturalistic reports and subjective accounts of the healing properties of artmaking, the body of scientific evidence from controlled research is still small [23], [24]. Ruddy and Milnes' systematic review identified 61 studies, but reported only two randomised controlled trials (RCT; total n = 137) comparing art therapy plus treatment as usual (TAU) with standard care alone in outpatients with schizophrenia or schizophrenia-like illness [8], which tentatively indicated positive effects of art therapy on self-esteem [12] and negative symptoms [25], but did not show any impact on psychosocial functioning or quality of life. Only one study was conducted in inpatients and reported positive effects of group art therapy compared to treatment as usual on social functioning [26]. In contrast, the largest and most recently published three-arm RCT of art therapy (MATISSE trial, [27]) in 417 patients with schizophrenia randomised to art therapy or an activity control intervention plus TAU or TAU alone, could not prove any additional benefit regarding global assessment of functioning or positive and negative symptoms scores at 12 and 24 months. However, this study included stabilised outpatients and reported substantial problems of attendance - median level of attendance was 11 sessions in the AT group over a 1-year period - as well as very low group sizes that might have limited the interaction between participants. Short term outcome data were not recorded, so that benefits during or shortly after the intervention could not be registered. As a conclusion, the authors recommended to study the impact of AT delivered in inpatient settings [27]. Therefore, the main purpose of the presented pilot study was to investigate the feasibility of an efficacy trial of art therapy for inpatients with schizophrenia during acute psychotic episodes and to generate preliminary data on the effects of art therapy on psychotic symptoms and global functioning. On an exploratory basis, we aimed to elucidate the influence of art therapy on measures of mentalising like cognitive empathy and levels of emotional awareness, as well as on self-efficacy, quality of life and satisfaction with care. For this reason a single-blind, parallel-group RCT was conducted, comparing a 6 week-course of 90 minutes of art therapy twice weekly in addition to TAU with standard treatment alone in patients with schizophrenia during acute episodes that required hospitalisation.

## Methods

### 2.1. Ethics and Registration

The study was approved by the local ethics committee (Charité Universitätsmedizin Berlin, EA1/104/11, positive vote: May 12, 2011) and conducted according to the principles expressed in the Declaration of Helsinki; all subjects gave written informed consent. Capacity to consent was assessed according to the principles defined by Helmchen and Lauter [28]. No patients who were unable to consent were included. In addition, legal representatives of patients, if present, were informed about study participation. The trial was registered at www.ClinicalTrials.gov (identifier: NCT01622166). Due to technical reasons, the study was registrated after enrolment started. The authors confirm that all ongoing and related trials for this intervention are registered. The protocol for this trial and supporting CONSORT checklist are available as supporting information; see **Protocol S1** and **Checklist S1**. The study started later and recruitment took substantially longer than expected at the time when the protocol was prepared.

### 2.2. Participants

Between 8/2011 and 9/2012, 122 inpatients of the Charité Universitätsmedizin Berlin (Psychiatric University Hospital at St. Hedwig Krankenhaus), aged between 18 and 64 years and diagnosed with schizophrenia according to DSM-IV, were screened and recruited by the treating psychiatrists with structured interviews (Structured Clinical Interview I and II (questions 103–117 for antisocial personality disorder) for DSM-IV, [29]) in order to exclude axis-I disorders other than schizophrenia and antisocial personality disorder. Other exclusion criteria were insufficient German language competence, any other psychiatric disorder apart from schizophrenia, relevant use of alcohol or illegal substances, organic brain disease or severe somatic disease impairing cerebral function, inability to give informed consent, acute suicidal intention, aggression and hospitalisation according to Berlin Mental Health Law (PsychKG). Patients were also excluded, when treatment in a group setting was impeded by 1) severe psychomotor agitation or impulsivity, 2) marked hostility and aggression, 3) severe paranoid ideation related to the setting or group members. Diagnoses according to DSM-IV-TR comprised paranoid (n = 45), disorganised (n = 4) and undifferentiated schizophrenia (n = 5), and first episodes of schizophreniform disorder (n = 4). Distribution of diagnoses did not differ between groups - neither in randomised patients nor in completers. Patients who were professionally involved in the making or the reception of art, or had attended art therapy as an outpatient before admission, were not included. Moreover, patients were asked to rate both their active and passive interest in art on a 4-point Likert scale (not at all - occasionally - regularly - passionate). There were no differences between groups, neither at randomisation nor in the per-protocol sample, and none between completers and dropouts.

### 2.3. Randomisation, concealment and masking

Fifty-eight patients were randomised using a randomisation list provided by the Charité central pharmacy. The allocation sequence was concealed until assignment by telephone. Participants and clinical staff involved in the administration and supervision of art therapy (D.S., L.H., K.D., U.H.) were aware of allocation status. Clinical ratings and scoring of Levels of emotional awareness scale (LEAS) transscripts were performed by psychiatrists (M.B., C.M.) not involved in the direct treatment of patients and blind to randomisation. Patients were instructed not to inform the assessors of their allocation status. Neuropsychological tests and questionnaires were administered by a researcher aware of therapy assignment (L.H.).

### 2.4. Interventions

Art therapy was administered in 12 sessions of 90 minutes for 6 weeks. Groups included between 3 and 6 patients. The setting was a room designed as an art studio in the clinic. It contained working tables, a variety of art materials such as water colour and poster paint, pencils, color pencils, markers, crayons, pastels, different size paper and brushes, drawing boards, clay, tools, an easel and storage facilities. The approach was non-directive – patients could choose to create whatever they wanted and use any available material they liked. They were encouraged to find their own image at their own pace. Interventions by the art therapist aimed at supporting the art process and helping to understand the image. The last 30 minutes of a session were reserved for a shared viewing and reflecting on the images. Each patient was asked if he/she wanted to present his/her image and to talk about it. Other patients were invited to comment and share their observations and ideas. If a patient did not want to share his image, this wish was respected. The patient’s autonomous decision-making about the handling of his or her art work was crucial. For a thorough description of therapeutic attitude, specific interventions and their rationale we refer to Dannecker (2010 [4]), serving as a therapeutic guideline for the art therapist.

One art therapist (D.S.) conducted the AT intervention and was accompanied by a co-worker who video-taped the sessions. All AT sessions were video-taped to assure treatment fidelity that was also confirmed during regular, joint video-based supervisions (K.D., U.H.), and to allow for qualitative analyses, which will be reported elsewhere. Patients assigned to the control group received TAU without an offer of art therapy. All patients were demanded to refrain from taking part in any other visual artistic activity apart from the sessions provided in the hospital during the trial phase. Patients who were discharged or transferred to a day clinic within the 12-session course were actively encouraged to attend all sessions. All patients received a designed reminder card of the session dates and were also motivated by personal contact and phone calls to participate.

Patients receiving TAU were also actively reminded of the follow-up examinations. In addition to supportive contact and pharmacotherapy, TAU covered a broad spectrum of verbal targeted psychotherapies for patients with psychoses like cognitive-behavioural therapy and modified psychodynamic interventions in individual and group settings, as well as occupational therapy, music therapy, cognitive and social skills training, excursions, relaxation and sports.

### 2.5. Outcome measures

Primary outcome measures were psychotic and depressive symptoms, measured by semi-structured interviews, which comprised composite scores of the Scale for the assessment of negative symptoms (SANS, [30]) and the Scale for the assessment of positive symptoms (SAPS, [31]), the Calgary depression scale for schizophrenia (CDSS, [32]), as well as psychosocial outcome, measured by the Global assessment of functioning scale of the DSM-IV-TR (GAF, [33]).

Secondary outcome measures were parameters of mentalising function, self-efficacy/locus of control, quality of life and satisfaction with care.

Mentalising was covered by the constructs of 1) cognitive empathy, determined with the Reading the mind in the eyes test (RME, [34]; German translation [35]) that requires the inference of complex affective mental states from photographs depicting the eye regions of 36 persons, and 2) the Levels of emotional awareness scale (LEAS, [36]; German version [37]), assessing the ability to imagine, to recognize and to express one's own and others' emotions when presented with 10 emotionally evocative written scenarios. Two parallel versions were used for testing at baseline and post-treatment visits in a balanced manner. The development of the LEAS was based on Piaget's model of emotional-cognitive development, which comprises 5 hierarchically ascending levels [38]. The increasing complexity and elaboration of emotional awareness of both own and others' emotional mental states is reflected by higher scores of the LEAS-self and LEAS-other scales, respectively, that were determined from transscripts of participants' answers using a detailed scoring manual and glossary. The 2 versions of the LEAS have shown a sufficient internal, inter-rater and test-retest reliability [37] and were also shown to capture changes in emotional awareness in the course of psychotherapeutic treatment [39]. LEAS-other scores correlated with tests of higher-order mentalising in patients with somatoform disorder [40]. From a first study in schizophrenic patients, Baslet et al. reported lower levels of emotional awareness of others in patients compared to healthy controls [41].

Self-efficacy and locus of control were evaluated by the Questionnaire for competence and control (Fragebogen zu Kompetenz- und Kontrollüberzeugungen, FKK, [42]), a translation and extension of Levenson’s IPC scales [43]. The FKK consists of 4 8-item scales, named self-concept of own ability, internality, social externality and fatalistic externality. Two secondary scales were calculated by adding two primary sub-scales and used for analysis: The self-concept of own ability and internality scales estimate global self-efficacy, and a sum score of the 2 externality scales reflects overall externality. Internality was found related to successful recovery in schizophrenia [14].

Quality of life during the past 4 weeks was evaluated using the core module of the Modular system for quality of life (MSQoL, [44]).

General satisfaction with hospital care was self-rated on an 8-item questionnaire (Fragebogen zur Patientenzufriedenheit, ZUF-8 [45]).

Potential confounders like verbal intelligence and verbal working memory and learning were estimated by a multiple choice vocabulary test (Mehrfachwahl-Wortschatztest, MWT-B, [46]) and the Auditory Verbal Learning Test (AVLT, [47]).

### 2.6. Procedure

Patients randomised to art therapy then were offered a trial AT session in which they were introduced to the setting and the circumstances of video-taping the sessions. Post-intervention, after approximately 6 weeks, a second examination was performed, including psychopathology, GAF, social-cognitive and emotional measures, locus of control and quality of life. Psychopathology, GAF, self-efficacy and quality of life measures were re-evaluated at a 12 weeks follow-up in both samples.

**Figure 1:**
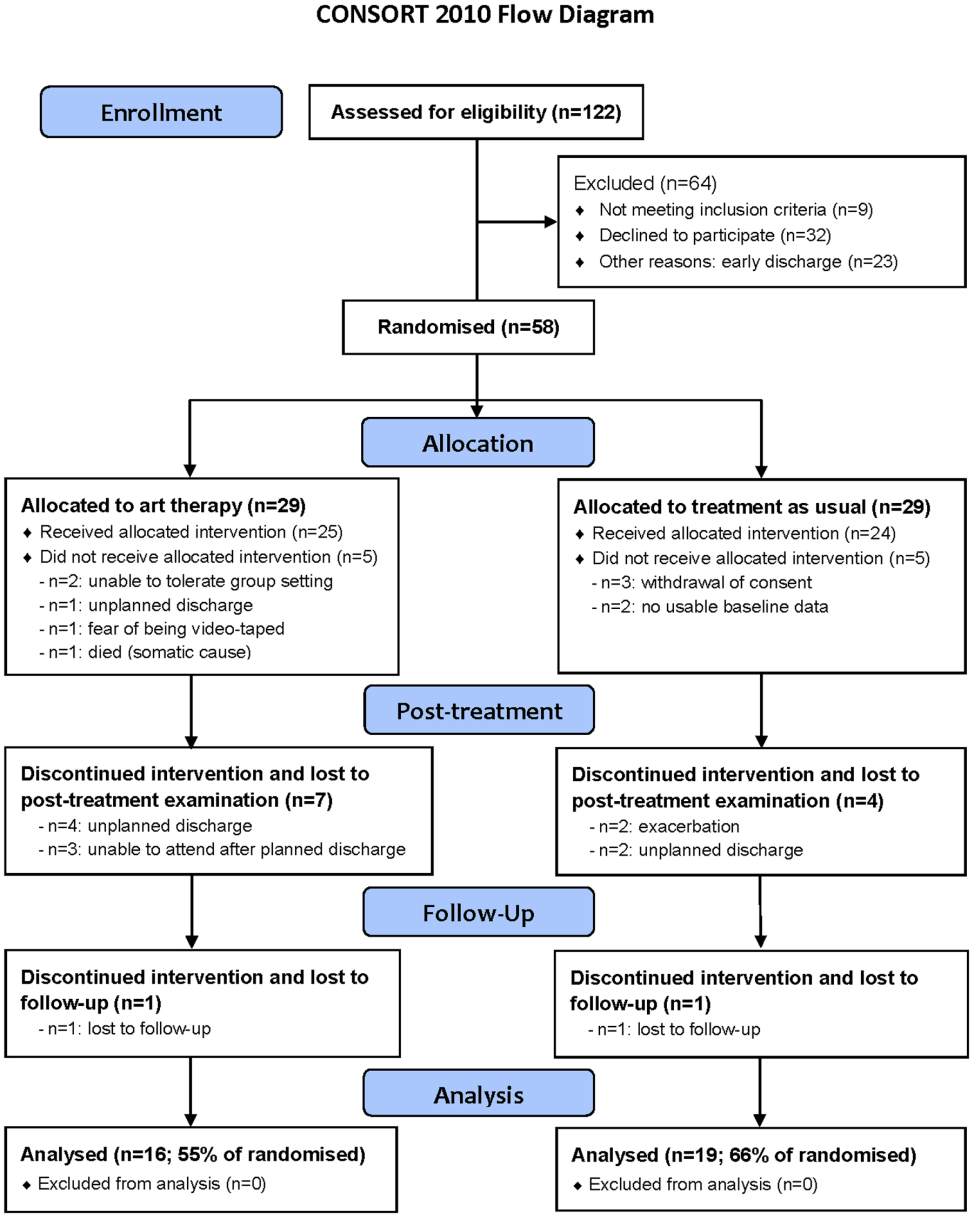
CONSORT 2010 flow diagram.

The progress through the phases of the trial of the two groups, as well as numbers and reasons for dropout are depicted in the CONSORT 2010 flow diagram (http://www.consort-statement.org/consort-statement/flow-diagram0/; **Figure 1**).

### 2.7. Statistical analyses

In this pilot study, no sample size calculation was performed. Statistical calculations were carried out as indicated in the results section using SPSS for Windows 20.0. All tests were performed with a 2-sided p<0.05. Data were tested for normality using the Kolmogorov-Smirnov-Test. The main analyses were performed on completer's data (per-protocol sample). T- and Chi^2^-tests were used to determine group differences of demographic and illness parameters. To account for possible regression to the mean and differences in baseline ratings, the primary outcome parameters SANS, SAPS, CDSS and GAF were compared between groups by ANCOVAs of both post-treatment and follow-up scores of SANS, SAPS, CDSS or GAF as dependent variables, intervention and gender as factors as well as the respective baseline scores and verbal IQ as covariates. Homoscedasticity was confirmed by Levene tests (p<0.05). Partial η^2^ values, reflecting the proportion of the total variance attributable to the intervention factor of the variance remaining after excluding variance explained by other predictors, are given as a rough estimate of effect size, with partial η^2^>0.14 representing small, η^2^>0.36 medium, and η^2^>0.51 large effects [48], p56. Secondary outcome parameters were analysed in the same manner. As social-cognitive function partly depends on neurocognition, AVLT^(1–5)^ scores were introduced as additional covariates for the group comparisons of social-cognitive measures. Group differences of overall satisfaction at post-treatment were determined by ANCOVA with the factors treatment group and gender, and the covariate verbal IQ. Multiple imputation was used to estimate missing post-treatment and follow-up scores to allow for an additional intention-to-treat analysis of primary outcome measures. Group differences were determined by mixed-model ANCOVA with intervention and baseline scores of the dependent variable as covariates. F-tests of 50 imputed data sets were pooled according to [49] using SPSS syntax by [50].

## Results

### 3.1. Sample characteristics and attendance

In total, 58 patients completed the baseline examination; 29 patients received art therapy and TAU; 29 patients were assigned to TAU. Twelve patients (1 female) left the AT group after an average of 2.2±1.8 sessions for reasons stated in **Figure 1**, five of which did not complete the introductory session. In 3 patients, the AT or trial setting was related to dropout (fear of video-taping, inability to tolerate group setting) - in all other cases reasons for dropout were unrelated to the setting. Nine patients (0 female) dropped out of the control group, 3 of which withdrew consent after being randomised to the control group. Two patients had no usable baseline data. Dropouts did not differ from completers in symptom severity at baseline, educational years or duration of illness. Only 3 patients completed less than 12 AT sessions, but 8, 8, and 9 sessions respectively. Two individuals were lost to 12-weeks follow-up. One patient died from a somatic cause unrelated to AT. No other serious adverse events were recorded.

Baseline demographic, illness characteristics and neuropsychological data are given in **Table 1**. When analysing completers only, patients in the AT + TAU group differed significantly from controls with regard to gender and verbal IQ at baseline. In the intention-to-treat sample, baseline SANS values in the AT group were significantly higher than in controls (T_[51]_ = −2.940, p<0.01; **Table 2**).

**Table 1:** Baseline demographic and neuropsychological parameters, and illness characteristics in patients receiving art therapy or treatment as usual.

	Per-protocol sample			Intention-to-treat sample		
	AT (n = 16)	TAU (n = 19)	Statistics	AT (n = 29)	TAU (n = 24)	Statistics
Gender (m/f)	7/9	15/4	**4.609** 1 *	19/10	19/5	1.206^1^
age (yrs)	38.8±11.9	39.6±10.6	0.231	37.4±11.2	38.8±10.4	0.473
education (yrs)	15.3±4.6	13.2±4.1	−1.438	14.2±4.6	13.7±4.0	−0.460
verbal IQ	116.6±14.5	105.5±13.5	**−2.339** *	107.2±17.7	108.6±14.5	0.314
AVLT ^(1–5)^	8.0±3.2	6.3±2.7	−1.655	7.4±3.0	6.6±2.9	−0.934
AVLT ^(int)^	3.3±2.7	3.6±2.6	0.428	3.0±2.4	3.6±2.4	0.940
duration of illness (yrs)	12.1±12.1	15.2±9.3	0.839	11.3±10.8	13.9±9.2	0.932
age of onset (yrs)	26.6±9.2	24.5±8.7	−0.713	25.6±7.6	24.4±8.8	−0.497
CPZ equiv. [mg]	352.5±264.1	522.0±298.4	1.763	451.0±362.7	501.8±326.2	0.531

T-test for independent samples;

1 : χ^2^-test;

*: p<005 (AT: art therapy; TAU: treatment as usual; AVLT: Auditory verbal learning test; CPZ equiv.: antipsychotic dose converted to chlorpromazine equivalents).

**Table 2:** Means, standard deviations, and group comparisons between art therapy and treatment as usual for psychopathological symptoms (SAPS, SANS, CDSS) and global assessment of functioning (GAF) in per-protocol and intention-to-treat samples at post-treatment and follow-up visits.

	baseline (0 wks)		post-treatment (6 wks)		follow-up (12 wks)		post-treatment (6 wks)	η^2^_p_	follow-up (12 wks)	η^2^_p_
	AT	TAU	AT	TAU	AT	TAU	AT vs.TAU		AT vs.TAU	
**per-protocol**	1	1	2	2	1	1	2		1	
SAPS	60.0±26.2	55.3±26.7	19.1±19.8	37.1±23.1	13.3±16.4	35.0±30.9	F_[1; 32]_ = 11.698**	**0.268**	F_[1; 30]_ = 6.960*	**0.188**
SANS	48.9±20.6	38.1±19.2	24.1±15.6	27.9±16.2	15.9±13.8	30.8±18.1	F_[1; 32]_ = 1.441	0.043	F_[1; 30]_ = 7.820**	**0.207**
CDSS	7.9±5.4	7.4±4.7	5.6±4.2	5.9±4.5	4.7±4.7	5.1±3.9	F_[1; 32]_ = 1.425	0.043	F_[1; 30]_ = 0.372	0.012
GAF	38.2±13.3	37.8±12.1	59.6±16.8	47.3±14.1	62.0±15.2	51.3±13.9	F_[1; 32]_ = 5.218*	**0.140**	F_[1; 30]_ = 5.005*	**0.143**
**intention-to-treat**	3	3	3	3	3	3	3		3	
SAPS	65.0±26.8	55.3±24.9	27.2±23.5	36.7±22.2	23.2±24.5	34.1±28.6	F_[1; 37.750]_ = 4,462*	-	F_[1; 37.349]_ = 2.858	-
SANS	53.9±22.0	37.3±17.4	27.6±16.8	27.1±15.5	24.6±19.1	29.3±17.4	F_[1; 39.205]_ = 0.768	-	*F_[1; 34.770]_ = 3.291* ^†^	-
CDSS	7.4±5.5	6.7±4.5	6.4±4.5	5.9±3.9	5.3±4.5	5.3±3.9	F_[1; 33.011]_ = 0.024	-	F_[1; 30.793]_ = 0.031	-
GAF	37.0±12.8	38.4±11.0	52.4±17.2	48.0±14.0	55.8±17.8	51.2±14.4	F_[1; 38.297]_ = 1.114	-	F_[1; 35.663]_ = 1.036	-

1 : n_AT_ = 16/n_TAU_ = 19;

2 : n_AT_ = 16/n_TAU_ = 20;

3 : n_AT_ = 24/n_TAU_ = 29.

Per-protocol sample: ANCOVA factors: intervention, gender; covariates: verbal IQ, baseline value of dependent variable; F_[df]_; *: p<0.05; **: p<0.01; η^2^_p_; corrected p-value (Bonferroni) p<0.0125 (significant results underlined).

Intention-to treat sample: descriptive statistics and ANCOVA on multiply imputed data (m = 50): covariates: intervention, baseline value of dependent variable; pooled F_[df]_; *: p<0.05; ^†^: p = 0.078 (AT: art therapy; TAU: treatment as usual; SANS: Scale for the assessment of negative symptoms; SAPS: Scale for the assessment of positive symptoms; CDSS: Calgary depression scale for schizophrenia; GAF: Global assessment of functioning).

### 3.2. Primary outcome measures

ANCOVAs with post-treatment and follow-up scores of SAPS, SANS, CDSS and GAF as dependent variables were performed, controlling for gender, verbal IQ and the baseline score of the respective dependent variable (**Table 2**). In the per-protocol sample, patients who had received AT had a significantly greater mean reduction of positive and negative symptoms at 12-week follow-up than patients treated as usual. Greater reductions of SAPS scores in the AT group already appeared at post-treatment and were maintained in the intention-to-treat analysis. The significance of the group difference in negative symptoms at 12 weeks was reduced to a trend. In the per-protocol sample, global assessment of functioning was significantly higher in the AT group at both visits, but not in the intention-to-treat sample. There were no group differences regarding depressive symptoms (**Table 2**), and were no significant interactions between intervention group and gender.

### 3.3. Secondary outcome measures

LEAS scores differed significantly between groups at post-treatment, favoring AT (LEAS-self: T_[34]_ = −2.186, p<0.05; LEAS-other: T_[34]_ = −2.596, p<0.05); however, after control for verbal IQ, AVLT^(1–5)^ and baseline values significance was maintained for LEAS-other scores only (**Table 3**). No group differences were detected for cognitive empathy at the Reading the mind in the eyes test. Patients receiving TAU showed significantly higher scores for generalised self-efficacy at post-treatment (T_[35]_ = 2.585, p<0.05) and at follow-up (T_[33]_ = 2.455, p<0.05), but this was not maintained at ANCOVA. There were no significant group differences in FKK generalised externality, quality of life and overall satisfaction with hospital care (**Table 3**).

**Table 3:** Means, standard deviations, group differences (F_[df]_) and effect sizes (η^2^_p)_ between art therapy and treatment as usual for social cognition (LEAS, RME), self-efficacy/locus of control (FKK), quality of life (MSQoL) and satisfaction with care (ZUF-8) scores in the per-protocol sample.

	baseline (0 weeks)		post-treatment (6 weeks)		follow-up 12 weeks)		post-treatment (6 weeks)	follow-up (12 weeks)
	AT	TAU	AT	TAU	AT	TAU	AT vs. TAU	AT vs. TAU
**per protocol**	1	1	1	1	2	2	1	2
LEAS-self 3	23.6±6.5	22.2±8.0	29.3±5.4	24.8±6.5		-	F_[1;29]_ = 2,129	-
LEAS-other^3^	18.9±6.8	19.4±9.2	26.9±6.5	20.6±7.9		-	F_[1;29]_ = 5.632*	-
RME^3^	20.5±5.5	20.6±5.9	22.3±4.3	20.7±5.5		-	F_[1;30]_ = 0.653	-
FKK SKI^3^	60.5±15.1	68.3±12.9	56.4±9.3	64.6±10.0	58.9±10.7	67.93±10.3	F_[1;30]_ = 1.646	F_[1;28]_ = 0.561
FKK PC^3^	62.4±18.2	60.7±13.5	55.6±13.0	54.4±16.5	52.4±10.8	57.5±13.2	F_[1;30]_ = 0.782	F_[1;28]_ = 3.989
MSQoL^4^	183.9±40.8	194.7±46.8	202.6±42.2	218,3±33.3	224.1±36.4	214.3±34.7	F_[1;31]_ = 0.098	F_[1;29]_ = 3.251
ZUF-8^4^		-	16.7±4.5	14.1±4.4		-	F_[1;31]_ = 1.522	-

1 : n_AT_ = 16/n_TAU_ = 20;

2 : n_AT_ = 16/n_TAU_ = 19;

3 : ANCOVA factors: intervention, gender; covariates: verbal IQ, AVLT^(1–5)^, baseline value of dependent variable.

4 : ANCOVA factors: intervention, gender; covariates: verbal IQ, baseline value of dependent variable; (post hoc) F_[df]_; *: p<0.05; corrected p-value (Bonferroni) p<0.007.

(LEAS: Levels of Emotional Awareness Scale; RME: Reading the Mind in the Eyes Test; FKK SKI: sum score self-efficacy; FKK PC: sum score externality; MSQoL: Modular system for quality of life; ZUF-8: satisfaction with care).

## Discussion

This pilot project aimed to investigate the feasibility of an efficacy study of art therapy in addition to TAU compared to stardard treatment alone in an assessor-blind, randomised controlled design in acutely psychotic, hospitalised patients diagnosed with schizophrenia.

### 4.1. Feasibility

Study design had to consider both the necessity of a relatively brief intervention period due to the shortness of an average hospital stay and the high initial symptom load of patients. Inability to consent and the cognitive demands of the baseline examination limited recruitment. Substantial numbers of screened patients were either discharged early or declined to participate for various reasons. Despite of this, recruitment and randomisation of participants was practicable. Future inclusion of an attractive, active control group might even facilitate recruiting, as patients were looking forward to the intervention. The non-pharmacological focus of the study was well-accepted, and helped to develop trust. Dropout occurred mainly in case of unplanned discharge from hospital or when patients were unable to attend the group as outpatients because of alternative occupations. Attendance of the AT group was good as soon as participants were engaged in the group and as long as they stayed in the hospital. At post-treatment, 59% of patients randomised to art therapy and 69% receiving TAU were examined, and 55% of patients treated with AT versus 66% of participants in the control group completed the follow-up examination at 12 weeks. Compared to the trials conducted in chronic outpatients, attrition rates of our acute patients tended to be higher. Green et al. reported 40% attrition at 20 weeks and 65% at 9-months follow-up [12], in the study of Richardson et al., 19% of patients had left at 12 weeks and 60% at 6-months follow-up [25]. Only 31% of the AT patients of Crawford et al. had attended 10 or more groups at 12 months [27]. Attrition rates can be considered as satisfactory, if the exacerbated state of the participants and the ubiquitous wish to leave the hospital as early as possible is kept in mind. (Unfortunately, the only other study of AT in inpatients was published in Chinese, and detailed data are not available [26]). Although statistically non-significant, a higher number of patients receiving AT than controls left the study early. Similar attrition rates across groups were found by Crawford et al. [27] and Richardson et al. [25], while Green et al. [12] reported higher attrition in the control group. Compared to these studies of stabilised outpatients, the greater demands of the AT sessions theoretically might have caused dropout of acutely psychotic patients, while patients in the control group had the chance to avoid intense therapeutic interactions if they wished to. However, only 3 out of 29 patients randomised to AT left early for study-related reasons. Otherwise the setting was well tolerated, and apart from one patient who died from a somatic cause that was unlikely to be related to AT, no adverse events were noticed in the AT group.

### 4.2. Primary outcome measures

As most patients were lost to follow-up after dropout, the main analysis of primary outcome parameters had to refer to the per-protocol sample. Results of this tentative analysis suggest a greater mean reduction of positive symptoms in patients receiving AT at post-treatment and at 12-week follow-up, even if baseline scores were controlled. Negative symptoms were lower in the AT group at follow-up. Results were partly maintained in the intention-to-treat analysis. There were significantly higher GAF mean scores in the AT group at post-treatment and follow-up, but no significant group differences as for depression ratings. Although in contrast to the methodologically rigorous MATISSE trial [27] that did not report any benefit of weekly AT at 12 and 24 months, our preliminary results point in a similar direction as findings of Richardson et al., who reported a reduction in SANS scores and better social functioning in 43 patients randomised to AT after 12 sessions and at 6 months follow-up compared to TAU (n = 47), and of Meng et al., who found an increase in overall mental health and social functioning associated with twice-weekly group art therapy over 15 weeks vs. TAU [25], [26]. Corroborating evidence from other creative therapies [51]–[53], our results support the notion that the arts therapies might be helpful in reducing negative symptoms in psychosis. Moreover, in the follow-up period SANS scores in the AT group further improved, while they deteriorated again in the control group. It therefore might be speculated that the experience of AT during acute psychotic episodes might possibly prevent an increase of negative symptoms after remission of acute psychosis.

The decrease of positive symptoms in the AT compared to the TAU group at post-treatment and follow-up should inspire further investigation: Our results contradict the notion that psychodynamic therapies may lead to high emotional intensities and deleterious aftermaths in psychotic patients [5], [54]. Our findings may correspond to subjective accounts of patients, who experienced stress reduction during AT sessions, and to the view that the AT setting and the artwork may “contain rather than stimulate anxiety” in psychotic patients [3], [5], [55].

### 4.3. Secondary outcome measures

Art therapy might counteract core negative symptoms like anhedonia, lack of motivation and affective blunting, but might also exert protective effects on self-esteem, self-efficacy and a creative sense of agency in the course of recovery. Beside a number of case reports and uncontrolled research [56]–[58], also Green et al. reported significant improvement of patients receiving AT in their attitudes towards themselves, which was correlated with self-esteem, and their ability to get along with others [12]. Meng et al reported higher values on the Tennessee Self Concept Survey, indicating that patients who received AT viewed themselves as more competent and valuable [26]. However, though externality scores nominally decreased in our AT group until follow-up, but did not so in controls, no significant influence of AT on externality or self-efficacy was determined in this study. Moreover, groups did not differ with regard to depressive psychopathology. Beside insufficient statistical power to detect minor effects in small-sized samples, findings might also indicate that the beneficial effects of AT in acute, compared to remitted psychoses might be associated with other mechanisms than an action on locus of control and depression.

Another exploratory focus of this study was directed to possible changes of mentalising abilities in the course of AT. Mentalising deficits, e. g. difficulties to recognize, to infer and to reflect about one's own and other's mental states have consistently been reported in schizophrenia [15], are related to symptom expression [19] and crucially linked to psychosocial outcome [59], [60]. While neuroscientific research tended to investigate the social-cognitive aspects of mentalising like theory of mind and social perception, psychotherapeutic approaches concentrate on mentalisation of affective experiences, its developmental origins and interplay with affect regulation [61], and have extended to the field of psychoses [62], [63]. While no group differences were detected for cognitive empathy [34] in our study, patients in the AT group showed higher levels of emotional awareness scores, in particular regarding others' emotions, at the end of the intervention period compared to patients treated as usual. This is interesting, as theoretical considerations suggest that AT may mediate between concrete and symbolic ways of thinking [64]. Although mental state reflection is an active ingredient of any form of psychotherapy and already targeted in a variety of therapies for schizophrenia [65]–[67], AT may bear additional benefits. Psychotic patients may externalise their inner states in a concrete physical way - the artwork than allows to reflect upon, to clarify and to re-internalize meaning of internal states, and to develop a mentalising stance; the intentional use of symbolic representations is encouraged. Self-exploration and insight are facilitated together with a strengthening of a creative sense of self [56], thus preventing self-stigma. Moreover, art-sharing and communication with the art therapist and group members may help to appreciate the perspective of others [4], to find a language for mental content and thus to facilitate explicit mentalising [13], [68]. This process may find its reflection in the levels of emotional awareness concept inspired by Piaget [69]. AT has been integrated in mentalisation-based treatment programmes for patients with Borderline personality disorder [70], in juvenile offenders [71] and was found to promote parental mentalisation [72]. Moreover, Kidd and Castano [73] showed that in healthy adults also the non-therapeutic engagement with works of art like literary fiction may positively influence theory of mind. The finding of more mature levels of emotional awareness after 6 weeks of AT might be associated with a recovery of the ability to reason about own and other's emotional mental states in psychotic patients. In contrast, no changes were detected for cognitive empathy. This might be explained by the fact that AT more intensely trains emotional awareness by introspection and imagination, compared to the ability to recognize emotions from facial expressions. However, our result is a preliminary indication from a first quantitative research attempt on the effects of AT on mentalising that requires further substantiation.

Our results do not indicate effects of AT on quality of life or overall satisfaction with hospital care. Previous findings have been inconsistent, showing positive [26] or no impact [25], [27]. We believe that the intervention period in our study might have been too short to significantly improve quality of life and that satisfaction with hospital care did not differ between groups because of the host of alternative therapeutic offers in the TAU condition.

### 4.4. Limitations and recommendations for further reasearch

During the course of this pilot study, results of the largest RCT of AT in schizophrenia, the MATISSE trial, were published [27]. Along with negative findings regarding the effects of AT in psychoses, the authors mentioned a number of practical problems that might hamper interpretability of this trial. We therefore tried to address problems of feasibility in an inpatient setting, but also decided not to refrain from hypothesis-testing and to tentatively report first results, intending to motivate a continuation of research on AT for patients with psychoses.

Besides sample sizes, our small study suffers a number of limitations. There was no standardised active comparison condition to control for the unspecific effects of therapeutic contact and group dynamics. However, patients receiving TAU were engaged in other therapies and did not nessessarily suffer from less therapeutic attention. The total amount of (psycho-)therapeutic interventions in both groups should be recorded in future trials.

Even when focusing on patients with acute psychotic episodes, the follow-up period of 12 weeks was rather short, and the benefits of AT might have vanished in the longer run. A possible solution in a larger trial could be to start the intervention during a phase of hospitalisation and to continue the study without interruption in the familiar setting for a longer intervention and follow-up period.

It has also to be mentioned that AT might have “selected” patients who profited from this intervention. AT completers were more often female and of higher verbal intelligence. Although we statistically controlled for gender and IQ, creative activity might have particularly appealed to a subgroup of patients with a better a priori prognosis. A substantial share of excluded participants did not receive the allocated intervention. Three patients of the control group withdrew consent because they were not happy with the result of randomisation and the need to abstain from creative therapies. As psychotherapy research always has to consider personal preferences and setting, a larger RCT might include a run-in period of both AT and the control condition before randomisation to avoid selection effects [74]. However, similar to the exclusion of subjects who never received the intervention after randomisation, this will limit generalisability of findings [75].

Higher attrition rates might not be avoidable in patients with severe mental disorders, but for a sound intention-to-treat analysis outcome data should be collected from all participants regardless of whether they received the intervention. This might be achieved by closely involving outpatient services, telephone reminders, provision of transportation costs and financial incentives. A higher frequency of study visits might not only allow for registering short term changes, but also enhance motivation of patients. Reasons for drop-out and missing of visits and the respective patient characteristics should be systematically analysed as these might not occur completely at random. The problem of missing data, mainly of dropped out individuals, impeded intention-to-treat analysis which had to rely on substitution by multiple imputation. Moreover, multiple imputation methods might also lead to biased results if high amounts of missing values are substituted. Future research should try to minimise missing data and allow for sensitivity analysis [76], [77].

Our findings are based on small case numbers, and we are aware of the fact that data on efficacy from such a small trial are biased and might overestimate the impact of the intervention. Moreover, patients in the per-protocol sample differed - though not significantly - in SANS and SAPS ratings, so that significant effects might be a result of regression to the mean. We accounted for this problem by using ANCOVA including baseline scores as covariates. For the purpose of a pilot study four primary outcome measures were tested, only two of which remained significant after alpha-level adjustment. Results may therefore only give a very rough indication of the direction of the treatment effect but cannot serve to confirm or reject our hypotheses, and even results on feasibility might not generalise [78].

### 4.5. Conclusion

Evidence on the efficacy and effectiveness of AT in patients with schizophrenia is far from being conclusive and benefits might be limited to a subgroup of patients. Results of this pilot study suggest that RCTs of AT can be implemented in routine hospital settings for patients experiencing acute psychotic states. With all due caution, findings from this first European pilot RCT of psychodynamic AT in acutely psychotic inpatients prove the feasibility of similar projects and point to a possible positive effect of the intervention on psychotic symptoms, psychosocial functioning and the ability to mentalise emotions. These preliminary results must be substantiated by further independent research.

## Supporting Information

Checklist S1 **CONSORT checklist.**
(DOC)

Protocol S1 **Trial protocol, version 1.0/date 08.11.2010.**
(DOC)
