# Supplementary material for: A Pilot RCT of Psychodynamic Group Art Therapy for Patients in Acute Psychotic Episodes: Feasibility, Impact on Symptoms and Mentalising Capacity
Source: PLoS One. 2014 Nov 13;9(11):e112348. doi: 10.1371/journal.pone.0112348 (PMC4231093; doi:10.1371/journal.pone.0112348)
Supplement: Protocol S1 — Trial protocol, version 1.0/date 08.11.2010. (DOC) [file pone.0112348.s002.doc]

**Group art therapy for symptom reduction**

**for patients in acute psychotic episodes - a pilot randomised controlled trial**

(German title:

Kunsttherapie zur Symptomreduktion bei

akuter Schizophrenie – eine randomisierte

kontrollierte Pilotstudie

**Code: KUSS**

**Trial Protocol**

**version 1.0 / date 08.11.2010**

**Principal Investigators:**

**Dr. med. Martin Bayerl, Dr. med. Christiane Montag**

Charité University Medicine Berlin (Charité Universitätsmedizin Berlin), Department of Psychiatry and Psychotherapy, Campus Mitte (Psychiatric University Hospital at St. Hedwig Krankenhaus)

**and**

**Prof. Dr. phil. Karin Dannecker**

Weissensee School of Art (Kunsthochschule Berlin-Weißensee), Art Therapy Department

**Sponsor:**

Charité University Medicine Berlin (Charité Universitätsmedizin Berlin)

**Financial support:**

Weissensee School of Art (Kunsthochschule Berlin-Weißensee), Art Therapy Department

**Table of Contents**

[1 Synopsis (according to GCP-V § 7 (3) Nr. 19 in German language) 5](#__RefHeading___Toc388910238)

[1.1 Flow Chart 10](#__RefHeading___Toc388910239)

[2 Introduction 11](#__RefHeading___Toc388910240)

[2.1 Introduction and Background Information 11](#__RefHeading___Toc388910241)

[2.2 Rationale 12](#__RefHeading___Toc388910242)

[3 Trial Objectives and Purpose 12](#__RefHeading___Toc388910243)

[3.1 Trial Design Overview 13](#__RefHeading___Toc388910244)

[3.2 Trial Duration/Timetable 13](#__RefHeading___Toc388910245)

[4 Participant Eligibility 13](#__RefHeading___Toc388910246)

[4.1 Inclusion Criteria 13](#__RefHeading___Toc388910247)

[4.2 Exclusion Criteria 13](#__RefHeading___Toc388910248)

[4.3 Rationale for inclusion of minors or other persons not able to give informed consent 14](#__RefHeading___Toc388910249)

[5 Investigational therapy 14](#__RefHeading___Toc388910250)

[5.1 Description of the art therapy / trial intervention 14](#__RefHeading___Toc388910251)

[5.1.1 Side effects and possible adverse events 14](#__RefHeading___Toc388910252)

[5.1.2 Treatment schedule 15](#__RefHeading___Toc388910253)

[5.1.3 Treatment compliance 15](#__RefHeading___Toc388910254)

[5.1.4 Therapist adherence to study treatment 15](#__RefHeading___Toc388910255)

[5.2 Description of control intervention 15](#__RefHeading___Toc388910256)

[5.3 Concomitant treatment 15](#__RefHeading___Toc388910257)

[5.4 Procedures in case of emergency 15](#__RefHeading___Toc388910258)

[5.5 Blinding Procedures 16](#__RefHeading___Toc388910259)

[6 Trial design / plan 16](#__RefHeading___Toc388910260)

[6.1 Trial centre and trial investigator requirements 16](#__RefHeading___Toc388910261)

[6.2 Methods of subject screening and recruitment 16](#__RefHeading___Toc388910262)

[6.3 Methods of obtaining informed consent 16](#__RefHeading___Toc388910263)

[6.4 Methods of avoiding simultaneous enrolment in other trials 17](#__RefHeading___Toc388910264)

[6.5 Methods of assigning subjects to treatment groups (randomisation) 17](#__RefHeading___Toc388910265)

[6.6 Clinical examinations and trial-related deviations of clinical practice 17](#__RefHeading___Toc388910266)

[6.6.1 Laboratory assessments 18](#__RefHeading___Toc388910267)

[6.7 Schedule of trial duration, treatment and subject participation 18](#__RefHeading___Toc388910268)

[7 Risk-Benefit-Analysis 18](#__RefHeading___Toc388910269)

[7.1 Risks, side-effects, strains, advantages and disadvantages 18](#__RefHeading___Toc388910270)

[8 Termination and Subsequent Treatment 19](#__RefHeading___Toc388910271)

[8.1 Premature termination of subjects / discontinuation / withdrawal 19](#__RefHeading___Toc388910272)

[8.2 Premature termination of trial 20](#__RefHeading___Toc388910273)

[8.3 Follow up procedures and continuing medical care after (premature) termination 20](#__RefHeading___Toc388910274)

[9 Adverse Events 20](#__RefHeading___Toc388910275)

[9.1 Definition of Adverse Events (AE) concerning the trial 20](#__RefHeading___Toc388910276)

[9.2 Definition of Serious Adverse Events (SAE) concerning the trial 20](#__RefHeading___Toc388910277)

[9.3 Definition of Suspected Unexpected Serious Adverse Reactions (SUSAR) concerning the trial 20](#__RefHeading___Toc388910278)

[9.4 Assessment of intensity 20](#__RefHeading___Toc388910279)

[9.5 Assessment of causality 21](#__RefHeading___Toc388910280)

[9.6 Recording of (Serious) Adverse Events 22](#__RefHeading___Toc388910281)

[9.7 Reporting of Serious Adverse Events (SAE) and Suspected Unexpected Serious Adverse Reactions (SUSAR) 22](#__RefHeading___Toc388910282)

[10 Documentation 22](#__RefHeading___Toc388910283)

[10.1 Case Report Forms (CRF) 22](#__RefHeading___Toc388910284)

[10.2 Investigator´s Site File 22](#__RefHeading___Toc388910285)

[10.3 Archiving the Data at the Trial Centre 22](#__RefHeading___Toc388910286)

[11 Quality Management 22](#__RefHeading___Toc388910287)

[11.1 Monitoring 23](#__RefHeading___Toc388910288)

[12 Data Management 23](#__RefHeading___Toc388910289)

[12.1 Data Collection / Case Report Forms 23](#__RefHeading___Toc388910290)

[12.2 Data Processing 23](#__RefHeading___Toc388910291)

[12.3 Generation of Pseudonym 23](#__RefHeading___Toc388910292)

[13 Statistical Analysis 23](#__RefHeading___Toc388910293)

[13.1 Determination of Sample Size 23](#__RefHeading___Toc388910294)

[13.2 Statistical Analysis 24](#__RefHeading___Toc388910295)

[13.2.1 Hypotheses 24](#__RefHeading___Toc388910296)

[13.2.2 Definition of Population for Analysis 24](#__RefHeading___Toc388910297)

[13.2.3 Analysis of Primary und Secondary Objectives 24](#__RefHeading___Toc388910298)

[13.2.4 Assessment of Safety 24](#__RefHeading___Toc388910299)

[13.2.5 Interim Analysis and Objectives of Premature Discontinuation 24](#__RefHeading___Toc388910300)

[13.2.6 Statistical Methods 24](#__RefHeading___Toc388910301)

[14 Reporting 24](#__RefHeading___Toc388910302)

[14.1 Statistical report 24](#__RefHeading___Toc388910303)

[14.2 Final report 25](#__RefHeading___Toc388910304)

[14.3 Publication (policy) 25](#__RefHeading___Toc388910305)

[15 Administrative Procedures 25](#__RefHeading___Toc388910306)

[15.1 Legal requirements of the trial 25](#__RefHeading___Toc388910307)

[15.1.1 Institutional review board 25](#__RefHeading___Toc388910308)

[15.1.2 Patient information and informed consent 25](#__RefHeading___Toc388910309)

[15.1.3 Insurance 25](#__RefHeading___Toc388910310)

[15.1.4 Data protection 25](#__RefHeading___Toc388910311)

[15.2 Study Financing 26](#__RefHeading___Toc388910312)

[16 References 26](#__RefHeading___Toc388910313)

# Synopsis (according to GCP-V § 7 (3) Nr. 19 in German language)

| **Titel der Studie** | Kunsttherapie zur Symptomreduktion bei akuter Schizophrenie  – eine randomisierte kontrollierte Pilotstudie |
| --- | --- |
| **Art des Vorhabens** | Monozentrische, einfach-verblindete, randomisiert-kontrollierte Pilotstudie |
| **Leiter der klinischen Prüfung/**  **Hauptprüfer** | Prof. Dr. phil. Karin Dannecker  Kunsttherapie Berlin  Kunsthochschule Berlin-Weißensee  Schönstraße 90, 13086 Berlin  kdannecker@kunsttherapie-berlin.de  Dr. med. Martin Bayerl  Psychiatrische Universitätsklinik der Charité im St. Hedwig-  Krankenhaus  Große Hamburger Straße 5-11, 10115 Berlin  Tel: 2311 2964  Fax: 2311 2962  martin.bayerl@charite.de  Dr. med. Christiane Montag  Psychiatrische Universitätsklinik der Charité im St. Hedwig-  Krankenhaus  Große Hamburger Straße 5-11, 10115 Berlin  Tel: 2311 2969  Fax: 2311 2962  christiane.montag@charite.de |
| **Prüfzentren** | Charité Universitätsmedizin Berlin,  Klinik für Psychiatrie und Psychotherapie, Campus Mitte,  Psychiatrische Universitätsklinik der Charité im St. Hedwig-  Krankenhaus |
| **Hypothese** | **Haupthypothese:** Die kunsttherapeutische Intervention führt im  Vergleich zu einer Kontrollbedingung zu einer schnelleren und  nachhaltigeren Symptomreduktion.  **Nebenhypothesen:**  NH1: Kunsttherapie führt zu einer größeren Zufriedenheit der  Patienten mit der Behandlung  NH2: Kunsttherapie ist assoziiert mit höherer Lebensqualität.  NH3: Kunsttherapie ist verbunden mit einer Zunahme der  Selbstwirksamkeitserwartung.  NH4: Kunsttherapie ist assoziiert mit einer Verbesserung von  Mentalisierungsfunktionen wie der ToM und der Erkennung von  Emotionen in sozialen Situationen |
| **Fragestellung aufbauend auf wissenschaftlichem Hintergrund** | Ist Kunsttherapie zur Behandlung von stationär geführten  Patienten in der akuten/ subakuten Phase einer schizophrenen  Erkrankung wirksam im Vergleich zur Standardbehandlung  (treatment as usual, TAU)?   - In der stationären Behandlung exazerbierter oder ersterkrankter schizophrener Patienten sind neben der antipsychotischen psychopharmakologischen Therapie und supportiven ärztlich-psychotherapeutischen Behandlung weitere gezielte therapeutische Interventionen notwendig, um den Genesungsprozess zu fördern. Die meisten Kliniken bieten dafür ein therapeutisches Programm mit verschiedenen Verfahren wie z.B. Psychotherapie, Psychoedukation, Ergotherapie, Bewegungstherapie, Musiktherapie, Dramatherapie und Kunsttherapie an. Dabei ist die gesicherte Evidenz über die Wirksamkeit der unterschiedlichen Interventionen sehr gering. In dieser Studie soll die Wirksamkeit der Kunsttherapie, einer seit über 60 Jahren etablierten Behandlungsform, bei Patienten mit akuten schizophrenen Psychosen geprüft werden. - Bei Patienten, die unter desorganisiertem Denken, Wahn, Sinnestäuschungen, Ich-Störungen, affektiven und/oder psychomotorischen Symptomen leiden und dadurch in ihrer Lebensqualität, Leistungsfähigkeit und sozialen Interaktion stark beeinträchtigt sind, ist der Aufbau eines stabilen therapeutischen Bündnisses und eine Symptomreduktion mithilfe antipsychotischer Arzneimittel die Behandlungsmethode der Wahl. Dafür ist häufig ein stationärer Rahmen notwendig. - In den sechziger Jahren wurden in den USA und Großbritannien Studien zur Kunsttherapie bei Menschen mit psychischen Störungen durchgeführt, die den Prozess der Therapie untersuchten und die Behandlungsform entwickelten. Die Kunsttherapie wurde als Studienfach an internationalen und seit ca. 20 Jahren an deutschen Hochschulen etabliert. Von Beginn an wurden auch an Schizophrenie leidende Menschen mit Kunsttherapie behandelt. Erkenntnisse über die positive Auswirkung von praktizierter künstlerischer Aktivität bei schizophrenen Patienten, wobei es zu einer Reduktion subjektiven Leidens durch den Ausdruck von Gefühlen und neuer Einsichtsmöglichkeiten im Kunstwerk kommt, gehen bis ins neunzehnte Jahrhundert zurück. - In einem Cochrane-Review von 2005 haben Ruddy et al. [4] die Evidenz für die Behandlung schizophrener Patienten mit Kunsttherapie zusammengefasst. Dabei wurden Hinweise für die Wirksamkeit dieser Therapieform in zwei in eine Metaanalyse eingeschlossenen Studien [5; 6] beschrieben. Randomisierte kontrollierte Studien, die eine gut abgesicherte Aussage über die Wirksamkeit und Unbedenklichkeit erlauben würden, liegen jedoch bisher nicht vor. - Wissenschaftliches Begleitprogramm: Neben der Untersuchung der symptomatischen Wirksamkeit der Kunsttherapie soll die Studie dazu beitragen, neue Erkenntnisse über Wirkfaktoren der Kunsttherapie, wie sie z.B. in der Patient-Therapeut-Interaktion zu erwarten sind, zu erbringen. Um diese Prozessforschung betreiben zu können, planen wir jede Kunsttherapieeinheit auf Video mitzuschneiden. Videoaufnahmen sollen neben der Sicherung der Therapieadhärenz der Therapeuten zur Gewinnung von objektiven und beobachtbaren Informationen über die Interaktionen und Veränderungen zwischen Patient und Therapeut und Gruppe dienen. Diese Beobachtungen werden von nicht am Therapieprozess beteiligten Personen ausgewertet und in Beziehung zu den Beobachtungen des Therapeuten und seinen Therapieprotokollen gesetzt. Vergleiche sollen zu gesicherten Einschätzungen von Verhalten und Veränderungen der Patienten führen. |
| **Prüfmedikation / Behandlungsstrategie** | Die kunsttherapeutische Intervention findet zweimal wöchentlich in Gruppen mit bis zu fünf Patienten statt. Eine Einheit dauert jeweils 90 Minuten. Insgesamt werden zwölf Einheiten Kunsttherapie in sechs Wochen angeboten. Die letzten  Einheiten der Intervention können auch nach der Entlassung  aus der Klinik als poststationäre Therapiegruppe besucht  werden. |
| **Vergleichstherapie** | Standardtherapie (Treatment as usual) |
| **Studiendesign** | Es handelt sich um eine prospektive, randomisierte  kontrollierte Pilotstudie.Alle Patienten erhalten die indizierte psychopharmakologische und ärztliche Behandlung und können an den Therapien der Station uneingeschränkt teilnehmen. Lediglich der Umgang mit Kunst darf nicht gefördert werden (Museumsbesuche, kreatives Arbeiten mit Material). |
| **Zeitplan** | - Beginn der Rekrutierung: Januar 2011 - Erwartetes Ende der Rekrutierung: Juni 2011 - Erwartetes Ende der Studie: August 2011 |
| **Gesamtzahl Patienten** | Es ist geplant, 15 Patienten pro Studienarm einzuschließen. |
| **Einschlusskriterien** | - Diagnose einer Schizophrenie gem. DSM-IV - Alter 18-64 Jahre - Ausreichend deutsche Sprachkenntnisse - Schriftliche Einwilligung (DvH, ICH-GCP) |
| **Ausschlusskriterien** | - Organische Hirnerkrankung - Sonstige psychische Störung gemäß DSM-IV- Achse I - Antisoziale Persönlichkeitsstörung gemäß DSM-IV - Relevante schwere somatische Erkrankung - Relevanter Konsum oder von Alkohol und illegalen Drogen (Ausschluss durch Drogenscreening im Urin) - Unfähigkeit, Inhalt und Bedeutung der Studie zu verstehen und informierte Einwilligung schriftlich zu geben. - Aktuelle Fremdgefährdung, Suizidalität oder andere Selbstgefährdung. - Unterbringung gem. PsychKG - Mangelnde Eignung für gruppenpsychotherapeutische Verfahren: 1. stark ausgeprägte Antriebsstörung, Feindseeligkeit oder Aggressivität, 2. schwere Impulskontrollstörung, 3. schwerste paranoide Symptomatik. |
| **Dokumentationszeitpunkte** | Screeninguntersuchung (Visite -1)/ Randomisierung/ Baseline-Untersuchung (Visite 1, Woche 1)/ Untersuchung nach Interventionsende (Visite 2, Woche 6)/ Untersuchung zum Follow-up (Visite 3, Woche 12) |
| **Zielgrößen** | Primäre Zielparameter:   - Reduktion von psychotischen Positiv- und Negativ-Symptomen in SANS und SAPS-Score, - Depressivität (Calgary Depression Scale), - Psychosoziale Funktion (global assessment of functioning, GAF)   Sekundäre Zielparameter:   - Patientenzufriedenheit (Fragebogen für Zufriedenheit mit der stationären Behandlung (ZUF-8) - Lebensqualität (Modular System for Quality of Life) - Selbstwirksamkeitserwartung (Fragebogen zu Kompetenz und Kontrollüberzeugungen) - Mentalisierungsfunktionen (ToM: Reading the Mind in the Eyes Test); Gewahrsein von Emotionen in sozialen Situationen: Levels of Emotional Awareness Scale (LEAS)) |
| **Sicherheit** | Abfrage und Dokumentation unerwünschter Ereignisse zu jeder Visite. Im stationären Verlauf kontinuierliche Beurteilung und Dokumentation der Symptomschwere und etwaiger unerwünschter Ereignisse. Die Teilnehmer werden weiterhin aufgefordert, sich bei Auftreten von unerwünschten Ereignissen zwischen den Visiten umgehend zu melden. |
| **Abbruchkriterien** | Im Einzelfall:   - Suizidalität - Exacerbation der psychotischen Störung - Fremdgefährdung - Neuauftreten von Ausschlusskriterien - Unterbringungsbedürftigkeit nach dem PsychKG.   Insgesamt:  Sollten mehrere Patienten durch die Behandlung destabilisiert werden, und Suizide, Exacerbationen, Gewalt oder Unterbringungen nach dem PsychKG vermehrt auftreten, so kann die Studienleitung über den Abbruch der Studien entscheiden. |
| **Statistische Auswertung** | Die statistische Analyse in Bezug auf die Outcome-  Parameter soll mit Hilfe von T-Tests, ANCOVA und MANCOVA mit Meßwiederholung als Intention-to-treat-Analyse vorgenommen werden, wobei ausscheidende Studienprobanden in die Endanalyse einbezogen werden und die Gründe für das Ausscheiden angegeben werden. |
| **Präklinische Untersuchungen** | Ohne Relevanz |
| **Mögliche Risiken, Kontraindikationen, Maßnahmen, die bei eventuellen Zwischenfällen zu ergreifen sind** | Mögliche Risiken:   - Durch die Bearbeitung von Ängsten, inneren Konflikten oder paranoid belegten Themen während der therapeutischen Intervention könnte es zu einer temporären Destabilisierung des Patienten mit steigender Dynamik von Wahn, Halluzinationen und formalen Denkstörungen oder Depressivität kommen. - In einzelnen Fällen könnte die Gefahr von Fehlverhalten mit Gefährdung des Patienten selbst oder Dritter ebenfalls zunehmen. Mögliche Nebenwirkungen/ Komplikationen wären dann: Suizidalität, Suizidversuche, Suizid und Aggressivität.   Kontraindikationen: siehe Ausschlusskriterien (auch Neuauftreten von Ausschlusskriterien)  Maßnahmen, die bei eventuellen Zwischenfällen zu  ergreifen sind:   - Deeskalierende und stützende Intervention durch den Kunsttherapeuten. In der Regel sollte die Therapieeinheit nach einem Krisengespräch und eventuell einer kurzen Pause mit allen Teilnehmern zu Ende geführt werden. Bei schweren Erregungszuständen muss die Therapieeinheit für den betroffenen Patienten abgebrochen werden. In diesem Fall sowie bei Verdacht auf Suizidalität oder Eigen- und Fremdgefährdung muss der behandelnde Arzt sowie der Studienarzt informiert werden. Geeignete Schutzmaßnahmen werden durch den Arzt eingeleitet. |
| Risiko-Nutzen-Abwägung | Die Kunsttherapie birgt aller Erfahrung nach für die Teilnehmer  nur geringe Risiken, und diese können im stationären  Behandlungsrahmen minimiert werden. Sollte es zu Steigerung  ängstlicher Erregung, Anspannung oder Feindseligkeit und Aggressivität kommen, so wäre der Therapeut zu einer geeigneten Deeskalation in der Lage und könnte jederzeit umgehend auf ärztliche Hilfe zurückgreifen, wenn dies notwendig sein sollte. Bei geringer Gruppengröße und  erfahrenen Gruppenleitern sollte in den meisten Fällen ein  erfolgreiches deeskalierendes Eingreifen möglich sein. Der zu  erwartende Nutzen der Intervention überwiegt bei weitem die  Risiken. Ärztliche Hilfe und Hilfe von Krankenpflegekräften steht auf der Station ständig zur Verfügung. |

## Flow Chart

Screening

(all patients admitted to hospital, inclusion and exclusion criteria)

Patient information and

(written) informed consent

Study inclusion and baseline examination

Randomisation to either group art therapy (AT) or treatment as usual (TAU)

(via central pharmacy of the Charité University Medicine Berlin)

Intervention period (6 weeks)

(2 AT sessions of 90 minutes per week vs. TAU)

Post-treatment examination (at week 6)

Follow-up examination (at week 12)

# Introduction

## Introduction and Background Information

Schizophrenia is a serious mental disorder affecting up to 1% of individuals worldwide [7]. Despite considerable advances of treatment options, particularly regarding antipsychotic pharmacotherapy, about two thirds of patients develop a longstanding or recurrent course of the disease and have to face severe consequences with respect to their quality of life, vocational and social exclusion as well as multiple hospitalisations. A considerable share of patients experience permanent or recurrent symtoms like delusions and hallucinations or suffer from negative symptoms that limit their motivation and drive as well as hedonic experience. Beside pharmacological antipsychotic treatment, which is often linked to substantial risks and side-effects, psychotherapeutic approaches to schizophrenia have been increasingly given consideration in recent decades [8]. As a result, a substantial body of scientific evidence exists with regard to psychoeducation, family interventions, CBT, social skills training etc. [12].

The national guidelines on schizophrenia of the German Society for Psychiatry, Psychotherapy and Neurology (DGPPN) [1] recommend the application of various forms of psychotherapy.

Moreover, "creative therapies" like art therapy, music therapy, dance and drama therapies are broadly recommended without considering scientific evidence for the specific techniques.

Jones et al. emphasise in their 2010 Cochrane review [9] the general need of evidence from randomised controlled research regarding the psychotherapeutic treatment of schizophrenia. Of note, there is a striking lack of evidence on the efficacy of these approaches in patients experiencing acute episodes of the disorder and exhibiting a higher degree of thought disorder, difficulties to establish social contact or to express themselves verbally. In contrast, clinical experience suggests that the early use of psychotherapeutic interventions in this patient group might be of substantial benefit with respect to their ability to deal with the impact of symptoms and other illness-associated factors, to social interaction and to the mitigation of stigma. Here, creative therapies might be particularly efficient.

The putative advantages of art therapy are based on a number of theoretical assumptions:

Art therapy might have various modes of action, comprising the therapeutic relationship, the artistic process and the creator's relating to her/his work. From a psychodynamic point of view, art therapy can be considered a tripartite process, with the material/ artwork mediating the relationship between patient and therapist. As many patients in acute psychotic episodes are unable to directly establish social contact without being overwhelmed by fear, the non-verbal approach of art therapy and the mediating properties of the artwork allow for a subtile regulation of closeness and distance and a less frightening way of communication and self-expression. The interventions of the therapist are often experienced as less intrusive than in dyadic relationships, as they are mainly directed towards the creative process. They have a safe-guarding, encouraging and holding function in a situation of self-exporation. The physical qualities of the different materials may correspond to different inner reactions, feelings, memories and fantasies. Mental states may translate into visible and touchable forms, the reflection of which can help patients to regain orientation in their emotional world and foster self-reflection and emotional awareness. The experience of creativity and the production of coherently and objectively perceivable reality can furthermore strengthen self-esteem and prevent discouragement. Another important therapeutic ingredient is the group setting, allowing to take different perspectives, to jointly reflect and appreciate the pieces of art [2; 3; 10].

The current state of scientific evidence in art therapy in schizophrenia is reported by Ruddy et al. (2005) [4] in a systematic review. Only 2 out of 61 studies were RCTs conducted under sufficient methodological rigour. Green et al. (1987) [5] in their study of 47 outpatients with mixed diagnoses (50% participants with schizophrenia) investigated the effects of 10 sessions of art therapy every 2 weeks compared to patients receiving TAU. Patients receiving art therapy were reported to show improved social interaction and self-esteem and were more likely to complete the trial. Richardson et al. (2002) [6] compared 43 outpatients with chronic schizophrenia after a 12-week course of weekly art therapy with 47 patients treated as usual. Despite high drop-out rates the intervention group experienced a significantly greater reduction of negative symptoms at 6-months follow-up compared to controls. There was a trend for favourable effects of art therapy on social functioning, but not on quality of life and other symptom scales. A number of other studies were non-randomised or case-reports.

## Rationale

It can be concluded that preliminary evidence from outpatients with schizophrenia points to positive effects of art therapy regarding symptoms, self-esteem and social functioning. Art therapy represents a widely applied and clinically valuable form of psychotherapy for the treatment of mental disorders and in the rehabilitation of somatic disorders. However, there is only insufficient evidence for its efficacy and harmlessness regarding the treatment of patients with psychotic disorders from randomised controlled research.

# Trial Objectives and Purpose

This pilot randomised controlled trial will serve to generate preliminary data on the efficacy and safety of art therapy in patients in acute/ subacute episodes of schizophrenia. Results will allow to prepare a larger multi-centre RCT including the determination of sample-sizes.

**Primary objective** of the pilot study is to determine whether an adjunctive administration of art therapy in addition to TAU will lead to a more substantial symptom reduction in hospitalised patients with schizophrenia compared to standard treatment.

Primary endpoints are the Scale for the assesment of positive symptoms (SAPS) [17], the Scale for the assesment of negative symptoms (SANS) [18] and the Calgary depression rating scale for schizophrenia (CDSS) [19] as well as psychosocial outcome, measured by the Global assessment of functioning scale of the DSM-IV-TR.

**Secondary objectives** refer to the question, which other domains of experience and functioning may be impacted by art therapy. According to previous evidence and theoretical considerations about possible mechanisms of action of art therapy we chose the following constructs and

Secondary endpoints:

- General satisfaction with care (ZUF-8 [22; 23], a modification of the General Satisfaction Questionnaire [22]),
- Quality of life (Modular System for Quality of Life, MSQoL [11]),
- Self-efficacy/ locus of control (Questionnaire for competence and control: Fragebogen zu Kompetenz- und Kotrollüberzeugungen, FKK [20]),
- Mentalising capacity (1.: Reading Mind in the Eyes Test [29, 30]; 2.: Levels of emotional awareness scale, LEAS [34]),

**Exploratory analyses:**

- Qualitative and process-outcome analyses will be performed on an exploratory basis using footage of the art therapy sessions.

**The following data are documented as possible confounding variables:**

- Demographics (age, gender, education, genetic background, age and gender of siblings, marital status, housing, vocational status, assisted living/ community psychiatric care)
- Illness characteristics (year/ age of first manifestation, course of illness, number of episodes, number of psychiatric hospitalisations, course of antipsychotic treatment, non-pharmacological treatments, suicidality, familiy history
- Neurocognition (Multiple choice vocabulary test, MWT-B; Wisonsin card sorting test, WCST; Auditory verbal learning test, AVLT)

**Structured clinical interviews** for DSM-IV (SCID I and SCID II items referring to antisocial personality disorder) are performed to confirm inclusion and exclusion criteria with respect to diagnosis.

## Trial Design Overview

The study is planned and performed as a monocentric, randomised controlled pilot study with two parallel groups. The intervention group will be administered 12 sessions of psychodynamic art therapy (twice weekly over 6 weeks, 90 minutes per session), in groups of up to 5 patients, led by a trained art therapist experienced in the treatment of psychoses. The intervention will be aided and each sessions will be video-taped by a trained auxiliary person.

Patients randomisd to the control group will receive treatment as usual (TAU), including all indicated therapy options provided by the study centre. Allocation is open for patients, the art therapist, supervisors and all persons directly involved in the practical execution of the trial.

Psychopathological ratings (SAPS, SANS, CDSS, GAF) are performed by clinicians responsible for the study who are blind to randomisation status. In this pilot study, at least 15 patients will be included into each arm. The total individual trial duration is 12 weeks.

## Trial Duration/Timetable

The trial will be started on January 15, 2011 after a positive vote of the Institutional Review Board (Ethics committee of the Charité University Berlin). From this date, recruitment will take place and is expected to continue until June, 2011. Approximately 30 patients will be included.

The study will be finished after the follow-up phase of the last patient included. Data management and statistical analyses are planned for QIII/IV/2011, the final report for December, 2011.

# Participant Eligibility

## Inclusion Criteria

- Diagnosis of schizophrenia according to DSM-IV
- Age 18-64 years
- Sufficient German language competence
- Written informed consent (DvH, ICH-GCP)

## Exclusion Criteria

- Organic brain disorder
- Any other psychiatric disorder apart from schizophrenia according to DSM-IV- axis I
- Antisocial personality disorder asccording to DSM-IV
- Relevant somatic disorder imparing cerebral function
- Relevant abuse alcohol and illegal drugs (urine drug screen)
- Inability to understand and comply with the requirements of the study and to give written informed consent
- Inability to participate in a group psychotherapy setting:
  - massive agitation, hostility oder aggression
  - severe impulsiveness
  - severe paranoid ideation
- Current endangerment of others
- Current suicidal intent or massive tendency to self-harm
- Hospitalisation according to Berlin Mental Health Law (PsychKG)

## Rationale for inclusion of minors or other persons not able to give informed consent

Minors or patients being not able to give informed consent will not be included. In addition, legal representatives of patients will be informed about study participation.

# Investigational therapy

## Description of the art therapy / trial intervention

Art therapy will be administered in a group setting of up to 5 patients twice weekly. The twelve sessions of psychodynamic art therapy (twice weekly over 6 weeks, 90 minutes per session) are led by a trained art therapist experienced in the treatment of psychoses. The intervention will be aided and each sessions will be video-taped by a trained auxiliary person. Patients will chose freely the topic of their work and the materials they prefer, but may be supported in this process by the therapist encouraging patients to try out materials and techniques, by a choice of illustrated art books and postcards. The therapist may intervene only if the patient requires technical advice, needs encouragement or might become disappointed. At the end of each session materials will be put away, and the images will be presented to the group. Patients are free to decide whether they would like to comment on their work. In this phase, phenomenogical description is more important than interpretation. If the patient agrees, group members may also reflect on her/his image and reveal their own impressions and associations. The sessions are closed after the safe storage of the images in folders. The artworks are kept until the end of hospital treatment. At the time of discharge, patients take their folders home.

### Side effects and possible adverse events

The processing of anxiety, inner conflict or topics linked to paranoid ideation during therapeutic intervention could lead to a temporary destabilization of the patient with increasing dynamics of delusions, hallucinations and formal thought disorder or depression. Possible side effects/ complications would be exacerbation of psychotic symptoms, suicidal ideation, suicide attempts, suicide and aggression.

Adverse effects may also result from the treatment in a group setting. Hoffmann et al. therefore created a list of indications and contraindications for group psychotherapy (see also [24] and [25]). Participants should be able to adhere to the framework agreements (such as dates, duration). Psychotic symptoms of other patients might impose a burden on participants, and problems may arise from unfavourable group dynamics that might not always be resolved by the therapist. However, the specific technique of art therapy as well as the inpatient setting are suitable to recognise and minimise risks. Art therapy is less demanding with respect to the mental stability of patients compared to other group therapies, because it allows for a unique triadic interaction between patient, therapist and work of art. Art therapy offers a creative space that can bear protective properties. A lot of time is spent working silently, and adverse tendencies in group dynamics can be effectively controlled by the therapist. However, individual adverse reactions to the group setting or as a result of high emotional intensities evoked by self-exploration in the creative process can not be completely excluded.

### Treatment schedule

Patients are treated in groups of up to five people. In the pilot study twelve sessions of art therapy will be performed, offered twice weekly. A session lasts 90 minutes.

### Treatment compliance

In this pilot study, only inpatients will be included. Patients receive a reminder card with the dates of all 12 sessions and the necessary study examinations. The majority of art therapy sessions will take place in the context of inpatient treatment, but the last remaining sessions can be attended after discharge. Of note, study participation must not interfere with hospital discharge plans. Patients will be reminded and motivated to participate in every session. After discharge, patients might be reminded by telephone calls to participate in the art therapy sessions and follow-up examinations. Compliance is ensured by continuous documentation of session attendance.

### Therapist adherence to study treatment

Therapist adherence to the studied intervention is guaranteed by video-taping each session and video-based supervision.

## Description of control intervention

Patients randomised to the control group receive standard treatment (as usual).

## Concomitant treatment

All patients receive the indicated standard hospital care adapted to their personal needs.

Study participation does not lead to any alteration of the envisaged treatment. However, the following activities are not allowed for participants of both study arms during the trial period:

- additional art therapy,
- visits to art galleries and museums,
- visits to art education seminars,
- creation of pictorial or sculptural works during occupational or other therapies.

## Procedures in case of emergency

In case of an exacerbation of symptoms or severe adverse reactions associated with the study intervention, emergency measures can be taken at any time by the treating psychiatrist, the psychiatrist in charge and senior physicians of the hospital. Symptoms associated with the study intervention may comprise suicidal intention, self-harm, endangerment of others, material damage, increase in psychotic symptoms like desorganisation, delusions, hallucinations, psychomotor agitation or stupor, affective tension, anxiety or perplexity, increase in depressive symptoms, etc.

Should critical situations arise during an art therapy session, nursing staff can immediately assist. The patient may like to finish the session and should be accompanied back to the ward. In case of severe adverse reactions, the intervention has to be discontinued immediately. The treating psychiatrist or psychiatrist in charge will be informed in any case and has to decide about further necessary measures. The treating psychiatrist will also decide about the patient's ability to continue study participation in cooperation with the principal investigator of the study. All adverse and serious adverse events are documented in the case report file and reported to the principal investigator within 24 hours.

## Blinding Procedures

Blinding
Participants, art therapists and supervisors as well as clinical staff involved in the administration of art therapy and practical organisation of the study are aware of allocation status. Clinical ratings are performed by psychiatrists responsible for the study, but not involved in the direct treatment of the participating patients and blind to randomisation. Patients are instructed not to inform the assessor of their allocation status.

Methods of Unblinding and Discontinuation

Unblinding of the psychiatrists responsible for clinical study assessments is only allowed for safety reasons. Regular unblinding of the trial is planned at the end of the trial (last visit, last patient), after closing the database.

# Trial design / plan

see flow chart (chapter 2)

## Trial centre and trial investigator requirements

The Department of Psychiatry, Charité Universitätsmedizin Berlin, comprises two hospitals (Clinic for Psychiatry and Psychotherapy, CCM, Charitéplatz 1, 10117 Berlin) and Psychiatric University Clinic at St. Hedwig Hospital (PUK SHK), Große Hamburger Str. 5-11, 10115 Berlin). The site is experienced in clinical trials as well as the performance of basic scientific research in the field of schizophrenia. About 600 in- and outpatients with schizophrenia are treated by the trial site quarterly. Experience from previous studies indicates feasibility to recruit a number of 30 patients within 6 months.

## Methods of subject screening and recruitment

The study will recruit patients with schizophrenia during acute psychotic episodes that require hospitalisation. Patients are screened after admission for treatment to one of the general psychiatric wards of the hospital. The population to be screened is based on all patients with schizophrenia referred to the wards (approx. 250 cases/ year, including multiple admissions). These patients are screened observing inclusion and exclusion criteria. As only one intervention group will be run parallel in the pilot study, screening will be stopped after 5 consecutive patients being randomised to the art therapy arm, and re-opened, when treatment places become available.

Accessibility of the intended number of patients: From the approximate number of 250 patients per year it can be assumed that in five months about 100 inpatients with schizophrenia will be treated. A large number of patients of about 70% will meet the inclusion criteria. If about half of them could be expected to agree to study participation, the required number of 30 patients for both study arms will be achieved within the planned recruitment period.

## Methods of obtaining informed consent

After screening for inclusion and exclusion criteria, patients will be informed by their treating psychiatrist about the possibility of study participation at first. This should be preferably done in the first stage of the hospital stay, after completion of the admission procedure and necessary diagnostics and start of treatment. If the patient expresses approval or wishes further information, a psychiatrist responsible for the study will inform her/him about the trial. The information given covers in easily comprehensible terms the aims and organisational features of the study, nature and possible side effects of the intervention, the right to revoke previously given written consent and to withdraw from the study at any time without disadvantage and without having to give an explanation, an offer of more extensive information, the allocation of patients to different treatment groups and randomisation, obligations of the patients and the care-takers, data protection and insurance issues.

Written informed consent is given after a sufficient amount of time to read the Patient Information Form handed over, to discuss the issue with relatives, caregivers and the treating physician and to ask additional questions. If the patient gives informed consent, she/he and the study physician will sign the Informed Consent Form, by which the latter confirms that inclusion criteria are met. Moreover, inclusion and exclusion criteria are confirmed by use of a structured clinical interview for DSM-IV (SCID 1 and 2) [26].

## Methods of avoiding simultaneous enrolment in other trials

Patients are informed that participation in another study at the same time must be reported to the study physician. In inpatients, simultaneous participation in other trials can be easily excluded as the treatment process can be reviewed by study personnel. At the time of the study, no other clinical trials will be conducted at the site. Participation in studies and experiments without intervention of less than 2 days/sessions is possible, if the principal investigator decides that this does not interfere with the course of the KUSS study.

## Methods of assigning subjects to treatment groups (randomisation)

After provision of written informed consent the baseline examination is carried out. Than patients will be randomly assigned to treatment regimens via a randomisation list provided by the central pharmacy of the Charité according to the relevant Standard Operating Procedure. The allocation sequence will be concealed until assignment by telephone.

## Clinical examinations and trial-related deviations of clinical practice

Clinical examinations and symptom ratings, blood tests, pregnancy testing, urine drug screening and ECG belong to necessary routine examinations in schizophrenic patients. Patients with first manifestation of psychotic illness undergo extensive clinical diagnostics including MRI and EEG. Taking a detailed history of the patient and repeated diagnostic interviews also belong to clinical routine. Structured clinical interviews, personality and some of the neuropsychological tests are usually administered according to clinical indication in routine diagnostic procedures. However, the majority of tests and ratings (quality of life, self-efficacy, social cognition) are exclusively performed for scientific purposes. Randomised allocation of treatment is a deviation from clinical practice, the risk of which is minimised by additional visits and standardised ratings of side-effects.

**Table 1:** time schedule for study examinations

| **Instrument** | **Visit 0:**  **Pre-trial** | **Visit 1: Baseline** | **Randomisation**  **and 6 weeks**  **intervention**  **phase** | **Visit 2:**  **Post-treatment**  **(within 7 days after end of intervention)** | **Visit 3: Follow-up**  **(within 7 days after 12 weeks follow-up date)** |
| --- | --- | --- | --- | --- | --- |
| **Informed consent** | x |  |  |  |  |
| **Inclusion criteria** | x |  |  |  |  |
| **Demographics** | x |  |  |  |  |
| **SCID I** | x |  |  |  |  |
| **SCID II (APD)** | x |  |  |  |  |
| **SANS/ SAPS** |  | x |  | x | x |
| **CDSS** |  | x |  | x | x |
| **GAF** |  | x |  | x | x |
| **MWT-B** |  | x |  |  |  |
| **WCST** |  | x |  |  |  |
| **AVLT** |  | x |  |  |  |
| **ZUF-8** |  |  |  | x |  |
| **MSQoL** |  | x |  | x | x |
| **FKK** |  | x |  | x | x |
| **RME** |  | x |  | x |  |
| **LEAS** |  | x |  | x |  |
| **Medication dose** |  | x |  | x | x |
| **Documentation of adverse events** |  |  | continuously | x |  |
| SCID I: Structured clinical interview for DSM IV, axis I; SCID II - APS: Structured clinical interview for DSM IV, axis II, screening for antisocial personality disorder; SAPS: Scale for the assesment of positive symptoms; SANS: Scale for the assesment of negative symptoms; CDSS: Calgery Depression Scale for Schizophrenia; GAF: Global Assessment of Functioning; MWT-B: Multiple choice vocabulary test (Mehrfachwahl-Wortschatz-Test); WCST: Wisconsin Card Sorting Test; AVLT: Auditory Verbal Learning Test; FKK: Fragebogen zu Kompetenz- und Kontrollüberzeugungen; ZUF-8: General satisfaction with hospital care (Fragebogen zur Patientenzufriedenheit mit der stationären Behandlung), HAQ: Helping Alliance Questionnaire; MSQoL: Modular System for Quality of Life;  RME: Reading the Mind in the Eyes Test; LEAS: Level of Emotional Awareness Scale. | | | | | |

### Laboratory assessments

Laboratory assessments are not planned in this study.

## Schedule of trial duration, treatment and subject participation

Treatment duration for each individual: 12 weeks

# Risk-Benefit-Analysis

## Risks, side-effects, strains, advantages and disadvantages

Under ethical considerations, the study follows strictly an ‘add-on’ approach. All patients receive the usual standard treatment including pharmacotherapy and any other indicated psychological or adjunctive therapy. The overall amount of adjunctive treatment sessions can be adapted in the control group by participation in additional sessions of occupational or other therapies.

There are no systematic reports on the possible risks and adverse effects of art therapy in general or for its use in the treatment of schizophrenia. In contrast, adverse effects and risks of (group) psychotherapy have been focused increasingly in recent years. Hoffmann et al. 2008 [13] conclude that group dynamic processes may yield negative effects for individual participants. As earls as in the 1960ies, Bergin et al. [13] demonstrated deteriorations of health in about 10% of patients who underwent psychotherapy. According to Margraf et al. [16] three causal categories may be responsible for adverse reactions associated with the psychotherapeutic process:

1. failure or adverse effect of an adequate therapy

2. failure or adverse effect of an inadequate, unprofessionally conducted treatment

3. mismatch between personalities of patient and therapist.

Group therapies might endanger patients if groups are too large, if therapies have a too strongly disclosing/interpreting character and if confrontations between participants are not limited. Also, wrong indication and lack of guidance may increase the risk of adverse reactions [15]. Negative socio-dynamic processes like rivalry, aggression, submission or withdrawal that are not sufficiently contained and resolved by the therapist may have an unfavorable impact on individual patients.

However, despite of potential risks, group therapies are a broadly used and essential means of hospital and outpatient treatment.

In the current study, potential risk for participants must be minimised. Art therapy sessions will be held in small groups. The method itself reduces the probability of interpersonal conflict and confrontation. During the session, the art therapist will successively contact every participant, and thus divide the session in a number of short interaction sequences. Joint reflection of the images is guided by the therapist who safeguards an appreciative and respectful atmosphere. Only qualified art therapists experienced in the treatment of psychotic persons will lead the study sessions.

However, it cannot be excluded that art therapy may cause adverse effects in some participants or the entire group. Emotional processes evoked by this form of therapy may gain greater intensity and lead to destabilisation. In vulnerable individuals this may possibly lead to exacerbation of symptoms, suicidality or aggression. On the other hand it has to be considered that the avoidance of social contact and emotional self-exploration within a professionally led group therapy may have even more deleterious effects on patients. The broad clinical use of art therapy in patients with schizophrenia has given no indication of adverse effects but rather of considerable benefits of this form of treatment.

The remaining risk can be sufficiently controlled within the inpatient setting, permanent professional contact and regular documentation of the patients' condition. All members of staff and study personnel are experienced in the recognition of psychotic crises and the necessary interventions.

Since the expected individual risk is minimal and the expected benefit for the group of schizophrenia patients is high, the overall evaluation of the risk-benefit-ratio is positive.

However, if the occurrence of adverse effects of art therapy was reported, this would be of high clinical interest and consecutively influence routine treatment strategies.

Patients randomised to the art therapy group experience an individual advantage, as psychodynamic art therapy was not offered routinely at the study site in the past. For the same reason, the control group receiving treatment as usual will not be deprived of an indicated therapy. For the whole group of patients with schizophrenia the trial might result in the additional advantage to be offered an evidenced, effective and non-hazardous form of therapy in the future.

# Termination and Subsequent Treatment

## Premature termination of subjects / discontinuation / withdrawal

For the following reasons the patient may be taken out of the trial:

- personal wish of the participant
- withdrawal of consent
- loss of contact, move
- newly manifested exclusion criteria
- repeated adverse events
- serious adverse event
- significant violations of protocol

## Premature termination of trial

For the following reasons the trial may be terminated prematurely:

- decision of the PI with regard to a new risk-benefit-evaluation (e.g. occurrence of non-justifiable risks)
- new scientific findings which do not justify further continuation
- non-sufficient recruitment

The decision about premature trial termination will be made by the principal investigators.

## Follow up procedures and continuing medical care after (premature) termination

No particular scheme is recommended for treatment after the 12 weeks treatment period. Study participation will not have any influence on aftercare. All patients will receive standard treatment.

# Adverse Events

All adverse events reported during the study must be included in the analysis.

## Definition of Adverse Events (AE) concerning the trial

An adverse event is any untoward medical occurrence in a patient or clinical trial subject administered a medicinal product/intervention and which does not necessarily have a causal relationship with this treatment (according to Directive 2001/20/EC).

An AE can therefore be any unfavourable and unintended sign (including abnormal laboratory finding), symptom, or disease temporally associated with the use of a medicinal (investigational) product/intervention, whether or not considered related to the medicinal (investigational) product/intervention.

AEs also include: any worsening (i.e. any clinically significant change in frequency and/or intensity) of a pre-existing condition that is temporally associated with the use of the study drug/intervention; abnormal laboratory findings considered by the reporting investigator to be clinically significant; and any untoward medical occurrence.

## Definition of Serious Adverse Events (SAE) concerning the trial

A serious adverse event is any untoward medical occurrence or effect that at any dose results in death, is life-threatening, requires hospitalisation or prolongation of existing hospitalisation, results in persistent or significant disability or incapacity, or is a congenital anomaly or birth defect (according to Directive 2001/20/EC).

## Definition of Suspected Unexpected Serious Adverse Reactions (SUSAR) concerning the trial

A Suspected Unexpected Serious Adverse Reaction (SUSAR) is an adverse event, the nature or severity of which is not consistent with the applicable product information, i.e. “expected” AE.

## Assessment of intensity

The assessment of severity will be determined by the investigator and recorded on the case report form (CRF) AE page and SAE form according to the investigator’s best clinical judgment, taking into consideration various factors such as the subject’s report, the physician’s observations, and the physician’s prior experience. The investigator will assess the individual (S)AE severity using the following scale:

| **mild:** | Event is easily tolerated and does not cause any real problem to the patient. It does not interfere with routine activities. |
| --- | --- |
| **moderate:** | Event interferes with routine activities. |
| **severe:** | It is impossible for the patient to perform routine activities. |

## Assessment of causality

The determination of the likelihood that the study intervention caused the AE (causality assessment) will be provided by the investigator. The assessment of causality will be determined by the investigator and recorded on the CRF AE page and SAE form according to the investigator’s best clinical judgment. The following questions should be considered in determining the relationship between the study intervention and the AE:

- Was the study intervention administered?
- Was there another obvious cause of the AE?
- What was the temporal sequence of the onset of the AE relative to the study intervention?
- Was the AE consistent with the known pattern of response to study intervention?
- Could the subject‘s clinical state, environmental or toxic factors, or other modes of therapy administered to the subject have caused or modified the AE?
- Did the AE resolve or improve after study intervention was discontinued or reduced?
- Did the AE remain the same or worsen after study intervention was discontinued or reduced?
- If the subject was re-exposed (rechallenged), did the AE recur or worsen upon re-exposure? Did the AE not recur or improve with re-exposure? (Clinical judgment should be used for determining if rechallenge is appropriate.)

Taking into account any of the above factors, the Investigator will assess the causality of the individual AEs using one of the following:

| **unassessable:** | Due to insufficient information the causality cannot be assessed. |
| --- | --- |
| **unlikely:** | The adverse event may easily be explained with sufficient other information that there is no causality with the study intervention. |
| **possible:** | The adverse event follows a reasonable temporal sequence after administration of the intervention or a known response pattern to the suspected intervention. It could readily have been produced by the patient´s clinical state, environmental or toxic factors, or other modes of therapy administered to the patient. |
| **probable:** | The adverse event follows a reasonable temporal sequence after administration of the intervention or a known response pattern to the suspected intervention. It could not be reasonably explained by the known characteristics of the patient´s clinical state, environmental or toxic factors, or other modes of therapy administered to the patient. It disappears or decreases on cessation or reduction in dose of study intervention. |
| **certain:** | The adverse event follows a reasonable temporal sequence after administration of the intervention, disappears after cessation or dose reduction of the intervention and occurs again on reexposition. |

## Recording of (Serious) Adverse Events

The investigator will closely monitor each patient for evidence of intolerance of the study intervention. All adverse events occurring during the course of the study must be reported on the appropriate pages of the Case Report Forms and followed up until they have disappeared.

## Reporting of Serious Adverse Events (SAE) and Suspected Unexpected Serious Adverse Reactions (SUSAR)

**SAE:**

All serious adverse events will be reported immediately to the sponsor by the investigators according to GCP-V § 12. SAE which need not be reported: surgeries or other inpatient residences known *before* inclusion in the trial. Serious adverse events which occur after conclusion of the study and can still reasonably be considered to be related to the study intervention are also to be reported. Any death during the study has to be reported to the sponsor within 24 hours. In cases where a pathologist's report may substantially assist in clarifying the event an autopsy should be performed. In addition biopsy materials, blood samples and specialist's reports should be provided if considered useful.

**SUSAR:**

The sponsor must report all suspected serious unexpected adverse reactions occurring during the course of the study to the Ethics Committee, Competent Authority (if applicable) and investigators within 15 days, according to GCP-V § 13. In case of death, the SUSAR has to be reported within 7 days, and further relevant information has to be provided within the next 8 days.

All transferred data have to be pseudonym. Reporting of SUSARs require unblinding of the individual.

# Documentation

## Case Report Forms (CRF)

All assessed data will be documented in paper CRF.

## Investigator´s Site File

All essential documents, such as signed informed consents, will be filed in an Investigator´s Site File and archived for at least 10 years (according to ICH GCP chapter 8).

## Archiving the Data at the Trial Centre

According to GCP guidelines, the sponsor ensure that all study documents such as the study files and patient documents are archived for at least 10 years after the end of the study or its premature termination. Administrative documents (communication with ethics committee, copies of protocol, amendments, patients' identification log, signed informed consent forms) will be archived for 10 years at the site. Data will only be deleted with the authorisation of the sponsor. All data and documents will be made available if requested by relevant authorities. Patient medical records will be archived according to current regulations (Krankengeschichtenverordnung, KgVO) for at least 15 years.

# Quality Management

All investigators will perform the study according to the study protocol. Protocol deviations will be documented with reasons. Regular meetings will be held to discuss the trial progress.

## Monitoring

There will be no external monitoring for this pilot study.

# Data Management

All data will be documented and saved in pseudonym form. Each patient is identified by a study patient ID, neither including the full birth date, nor initials. The investigators document the allocation of the patient's name to the ID in a patients' identification log. Only the principal investigators, study physicians and other study responsibles have access to this log.

## Data Collection / Case Report Forms

Data will by collected via paper-CRF forms (CRF). CRFs will be stored separately from the patients' identification log. CRFs will be filled in by ball-pen, pencils are not allowed. Corrections can be made by crossing out an entry, correct onformation is given directly next to the previous entry, the study responsible/physician will sign the correction with his name, date and reason for correction. Data fields left open due to missing information have to be commented on.

The CRFs will be archived for 10 years.

## Data Processing

After form reading and data export, data management includes plausibility, consistence and range checks with possible following data corrections. Non-plausible or missing entries can be corrected in accord with the study physician; correction protocols will be stored together with the CRF.

The validated data base will be saved as "KUSS_date". The clear data base will be called as “closed”. The logging of all steps of data management procedures and corrections will be saved in (repeatable) scripts for PASW 18.0.

## Generation of Pseudonym

The pseudonym will neither contain initials nor birth-dates, and is composed as follows: "KUSS_randomisation number".

# Statistical Analysis

## Determination of Sample Size

No usable effect sizes are reported in the literature as a necessary basis for sample size estimation. Therefore, no sample size estimation will be performed. This pilot trial is conducted to generate data and to estimate effect sizes to prepare for a future, larger efficacy trial.

## Statistical Analysis

### Hypotheses

**Primary Hypothesis:**

- HH: The administration of 12 sessions of psychodynamic group art therapy in addition to standard treatment is associated with a greater reduction in positive, negative and depressive symptoms and with better psychosocial functioning compared standard treatment alone.

**Secondary Hypotheses:**

The administration of 12 sessions of psychodynamic group art therapy in addition to standard treatment is associated with....

- NH1: higher overall satisfaction with care,
- NH2: higher quality of life,
- NH3: a higher expectation of self-efficacy and a more internal locus of control,
- HN4: higher measures of cognitive empathy and emotional awareness, i. e. increased mentalising ability

### Definition of Population for Analysis

The intent to treat analysis will be executed with all available patient’s data independent of dropouts and protocol violations. The per protocol analysis will be accepted only for the patients who finished the trial in accordance with the study protocol (valid cases).

### Analysis of Primary und Secondary Objectives

The primary objective is to determine differential changes of symptom ratings (SANS, SAPS, CDSS) and psychosocial function (GAF score) from baseline. Measurement points are the examinations post-treatment (6 weeks) and at follow-up (12 weeks). Changes are compared between intervention and control groups under consideration of possible confounding variables (repeated measures ANOVA/ ANCOVA, two-tailed level of significance: alpha=0.05).

Secondary objectives and the respective endpoints (satisfaction with care, self-efficacy, social cognition) will be analysed in the same manner. Statistical calculations will be carried out using PASW 18.0.

### Assessment of Safety

The safety analysis includes all patients with at least one session of art therapy. Safety parameters will be analysed descriptively.

### Interim Analysis and Objectives of Premature Discontinuation

An interim analysis is not performed.

### Statistical Methods

See chapter 13.2.3

# Reporting

## Statistical report

The PI will perform the statistical analysis and will prepare the final clinical trial report (CTR). This final CTR will be released by all colloborators.

## Final report

The integrated final report will be written by the PI according to ICH E3 and includes the statistical report.

## Publication (policy)

It is understood that all results concerned with this study will be maintained in confidence.

The results of the study are published at scientific meetings or in a journal after appropriate consultation irrespective of the outcome and by mutual agreement between all investigators and collaborators.

# Administrative Procedures

## Legal requirements of the trial

The clinical trial described in this protocol will be conducted in compliance with the Declaration of Helsinki, ICH guidelines for GCP and applicable local regulatory requirements and laws. During the pre-trial activities, the investigators and their staff must have obtained/confirmed a working knowledge of the regulatory requirements applicable to the clinical trial. In particular, the investigators must be aware of their responsibilities, as described in Chapter 4 of the ICH guideline for GCP and in compliance with other applicable regulatory requirements.

### Institutional review board

It is the responsibility of the investigator to obtain prospective approval of the trial protocol, protocol amendments, and other relevant documents, e.g., advertisements, if applicable, from the IRB (ethics committee of the Charité University Medicine, Berlin). The Clinical Trial will not begin before having the approvals of the IRB.

### Patient information and informed consent

Patient information: Before inclusion in the trial, patients will be informed about the aims, methods, characteristics, advantages, possible risks and side effects be the study physician. A Patient Information Form will be handed over to the patient. Patients will be given sufficient time to reflect their decision to participate.

Informed consent: Patients must declare their consent to participate in the trial and sign the Informed Consent Form. Patients receive a copy of both forms, the other copies is archived at the study site. Patient Information and Informed Consent Form have to be approved by the IRB (see chapter 6.3.)

### Insurance

Insurance will take out automatically for all patients who are enrolled in the study to cover AEs and any harm caused by the trial intervention or related to the study procedures. The insurance will be provided by a local insurance company.

### Data protection

Patients will be informed that their data will be saved under pseudonyms and used for scientific applications, congress reports and publications. Patients are entitled to be informed about the archived data. Patients are also informed that their data might be transmitted to the local ethics committee. Patients who do not consent to this requirement, cannot be enrolled in the study.

## Study Financing

The study is partially supported by the Weissensee School of Art.

# References

1. Deutsche Gesellschaft für Psychiatrie, Psychotherapie und Nervenheilkunde DGPPN (2005) S3 Praxisleitlinien in Psychiatrie und Psychotherapie. Band 1 – Behandlungsleitlinie Schizophrenie. Steinkopff-Verlag, Darmstadt
2. Dannecker, K, (2006/2010), Psyche und Ästhetik. Die Transformationen der Kunsttherapie. Berlin, MWV
3. Dannecker, K.. ( 2006) Fragemntierung im schizophrenen Erleben – ein Beitrag aus der Kunsttherapie, Praxis klinische Verhaltensmedizin und Rehabilitation 73:204-212
4. Ruddy R, Milness D (2005) Art therapy for schizophrenia or schizophrenia-like illnesses. Cochrane Database Syst Rev. 2005 Oct 19;(4):CD003728
5. Green BL, Wehling C, Talsky GJ (1987) Group art therapy as an adjunct to treatment for chronic outpatients. Hospital and Community Psychiatry 38(9):988–91
6. Richardson P, Jones K, Evans C, Stevens P, Rowe A (2002) An exploratory randomised trial of group based art therapy as an adjunctive treatment in severe mental illness. Supplied by author.
7. Jablensky A (1995) Schizophrenia: recent epidemiologic issues. Epidemiol Rev. 17(1):10-20
8. Kane JM, Correll CU (2010) Pharmacologic treatment of schizophrenia. Dialogues Clin Neurosci. 12(3):345-57
9. Jones C, Cormac I, Silveira da Mota Neto JI, Campbell C (2004) Cognitive behaviour therapy for schizophrenia. Cochrane Database Syst Rev. 2004 Oct 18;(4):CD000524
10. Killick, K, Schaverien, J (1997) Art, Psychotherapy and Psychosis London, Routledge
11. Pukrop R, Schlaak V, Möller-Leimkühler AM, Albus M, Czernik A, Klosterkötter J, Möller HJ (2003) Reliability and validity of Quality of Life assessed by the Short-Form nd the Modular System for Quality of Life in patients with schizophrenia and patients with depressio. Psychiatry Research 119:63–79
12. Pfamatter M, Junghan UM, Brenner HD (2006) Efficacy of psychological therapy in schizophrenia: conclusions from meta-analyse. Schizophr Bull. 2006 Oct;32 Suppl 1:S64-84
13. Hoffmann SO, Rudolf G, Strauß B (2009) Unwanted and damaging effects of psychotherapy An overview with a draft of a new model. Psychotherapeut 53:4–16
14. Bergin AE (1963) The effects of psychotherapy: negative results revisited. J Consult Psychol. 10:244–250
15. Roback HB (2000) Adverse outcomes in group psychotherapy: risk factors, prevention and research directions. J Psychother Pract Res. 9(3)113-22
16. Margraf J, Schneider S (2009) Lehrbuch der Verhaltenstherapie. Springer Medizin Verlag, Heidelberg
17. Andreason NC (1983) Scale of the assesment of positive Symptoms. University of Iowa
18. Andreason NC (1983) Scale of the assesment of negative Symptoms. University of Iowa
19. Müller MJ, Marx-Dannigkeit P, Schlösser R, Wetzel H, Addington D, Benkert O (1998) The Calgary Depression Rating Scale for Schizophrenia: development and interrater reliability of a German version (CDSS-G). Journal of Psychiatric Research 33 433-443
20. Krampen G (1991) Fragebogen zu Kompetenz- und Kontrollüberzeugungen (FKK). Hogrefe – Verlag für Psychologie, Göttingen
21. Andreasen NC, Pressler M, Nopoulos P, Miller D, Ho BC (2010) Antipsychotic dose equivalents and dose-years: a standardized method for comparing exposure to different drugs. Biol Psychiatry. 67(3):255-62
22. Schmidt J, Lamprecht F, Wittmann WW (1989) Zufriedenheit mit der stationären Versorgung. Entwicklung eines Fragebogens und erste Validitätsuntersuchungen Psychother Psychosom Med Psychol 1989; 39: 48-255
23. Schmid R, Neuer T, Cording C, Spießl H (2006) Lebensqualität schizophren Erkrankter und ihr Zusammenhang mit Krankheitsbewältigungsstrategien und Behandlungsaspekten. Psychiat Prax 33(7): 337-343
24. Burlingame GM, MacKenzie KR, Strauß B (2004) Evidence-based small group treatments. In: Lambert M (ed) Bergin & Garfield’s handbook of psychotherapy and behavior change. 5th edn. Wiley & Sons, New York
25. Strauß B, Eckert J (2001) Schäden und negative Folgen von Gruppenpsychotherapien. Gruppenpsychother Gruppendyn 37: 45–67
26. Wittchen HU, Zaudig M, Fydrich T (1997) SKID-I/II Strukturiertes Klinisches Interview für DSM-IV. Users guide for the structured clinical interview for DSM-IV - German modified version.
27. Siegfried Lehrl (2005) Mehrfachwahl-Wortschatz-Intelligenztest MWT-B. Balingen: Spitta Verlag, 5. unveränderte Aufl.
28. Computerised Wisconsin Card Sort Task Version 4 (WCST) Psychological Assessment Resources, 2004
29. Kettle JW, O'Brien-Simpson L, Allen NB (2008) Impaired theory of mind in first-episode schizophrenia: comparison with community, university and depressed controls. Schizophr Res. 99(1-3):96-102
30. Baron-Cohen S, Wheelwright S, Hill J, Raste Y,Plumb I (2001) The ‘‘reading the mind in the eyes’’ test revised version: A study with normal adults, and adults with asperger syndrome or high-functioning autism. Journal of Child Psychology and Psychiatry, 42, 241–251
31. Alexander LB, Luborsky L (1984) The Penn Helping Alliance Scales In Greenberg LS, Pinsof WM (Eds.) The Psychotherapeutic Process: A Research Handbook. New York, Guilford Press.
32. Rey RA (1941) L’Examen psychologique dans les cas d’encephalopathie traumatique, Archives de Psychologie 28: 286–340
33. Hawkins KA, Dean D, Pearlson GD (2004) Alternative forms of the Rey Auditory Verbal Learning Test: a review. Behav Neurol.15(3-4):99-107
34. Kessler H, Traue HC, Hopfensitz M, Subic-Wrana C, Hoffmann H (2010) Levels of Emotional Awareness Scale-Computer – Deutschsprachige digitale Version. Psychotherapeut 55: 329-334
